# Green Methodologies for Copper(I)-Catalyzed Azide-Alkyne Cycloadditions: A Comparative Study

**DOI:** 10.3390/molecules24050973

**Published:** 2019-03-10

**Authors:** Marissa Trujillo, Clayton Hull-Crew, Andrew Outlaw, Kevin Stewart, Loren Taylor, Laura George, Allison Duensing, Breanna Tracey, Allen Schoffstall

**Affiliations:** Department of Chemistry and Biochemistry, University of Colorado Colorado Springs, Colorado Springs, CO 80918, USA; mtrujil3@uccs.edu (M.T.); chullcre@uccs.edu (C.H.-C.); aoutlaw4@uccs.edu (A.O.); kstewar4@uccs.edu (K.S.); ltaylor6@uccs.edu (L.T.); lgeorge@uccs.edu (L.G.); allison.duensing@ucdenver.edu (A.D.); breanna.tracey@outlook.com (B.T.)

**Keywords:** 1*H*-1,2,3-triazole, copper-catalyzed azide-alkyne cycloaddition (CuAAC), microwave heating, solvent-free reaction, green chemistry, fluorotriazole

## Abstract

Successful copper(I)-catalyzed azide-alkyne cycloaddition (CuAAC) reactions may be achieved by several methods. In this paper, four synthetic protocols were performed for direct comparison of time required for the synthesis, yield, and purity of the 1*H*-1,2,3-triazole products. The methods with Cu(I) catalysts were conventional, microwave heating, solvent-free, and a method using glycerol solvent. The compounds synthesized in this paper were known non-fluorinated triazoles and new fluorinated triazoles. The results lead to the conclusion that the microwave method should be strongly considered for CuAAC syntheses.

## 1. Introduction

Recent applications of 1,4-disubstituted 1*H*-1,2,3-triazoles highlight the importance of methods for their synthesis [1,2], which are generally based upon the original reports of the copper(I)-catalyzed azide-alkyne cycloaddition (CuAAC) method [3,4]. The methodology developed since these groundbreaking reports includes syntheses with ligands for asymmetric synthesis [5], recyclable catalysts [6], Cu nanoparticles [7], complex catalytic systems [8], solid phase supports [9,10], various dipolarophiles [11], and microwave heating [12,13]. Microwave heating is used routinely for organic synthesis, although the theory and the reasons for its effectiveness are not thoroughly understood [14,15,16]. Microwave heating has proven to be useful for many organic reactions [17,18,19], including promotion of CuAAC reactions [20,21,22]. CuAAC reactions with microwave heating result in higher yields in less time than conventional heating [23].

Several green chemistry principles apply to the CuAAC reactions in this paper [24], which are important in synthetic organic chemistry [25] and in pharmaceutical process chemistry [26]. The CuAAC triazole syntheses have green solvents or no solvent, use minimal energy, are safe, and are catalytic. A green carbene catalyst is utilized in one of the four methods in this paper [27,28]. This N-heterocyclic carbene (NHC) catalyst is typical of those known to assist in C-C and C-N bond formation with potential for improving industrial processes, reducing waste, and causing less pollution [28,29]. We set out to determine which of the chosen methods stood out with respect to overall yield and purity of the product, as well as the time necessary to complete the synthesis.

The original reports of CuAAC reactions carried out in aqueous alcohol solvent are green methods with excellent atom economy [1,2]. The CuAAC method using CuI in glycerol similarly employs a green solvent [30]. Other recent methods not compared in the present paper employ synthesized recyclable catalysts. Some require special reaction conditions, but they offer efficient syntheses of triazoles in a short time, although longer than the microwave method [31,32,33,34]. Comparison of conventional and microwave methods for preparing 1*H*-1,2,3-triazoles have also been reported using copper salts with other catalysts [35,36].

Syntheses of fluorinated 1*H*-1,2,3-triazole derivatives are desirable for their potential biological activities [37]. The fluorinated triazoles are generally less polar than non-fluorinated derivatives. The present paper consists of the preparation of several new 1,4-disubstituted fluorinated 1*H*-1,2,3-triazoles using the same four reaction conditions as for the non-fluorinated triazoles.

## 2. Results and Discussion

### 2.1. Non-Fluorinated Triazoles

The four methods for preparing the triazoles in this paper are: (a) conventional (with or without heat) in alcohol/water solution with CuSO_4_/ascorbic acid; (b) microwave heating in alcohol/water solution using CuSO_4_/ascorbic acid; (c) solvent-free conditions using a copper carbene catalyst; and (d) room temperature conditions in glycerol using a Cu(I) catalyst. The green experimental conditions included alcohol or aqueous solvents, microwave heating, or solvent-free reaction conditions. The microwave method included devising a suitable procedure using the CEM Discover instrument. The alcohol/water solvent used for conventional heating was adopted for the microwave experiments to allow for direct comparison. The alcohol/water mixture was rated as having medium ability for absorbing microwaves [15]. It was determined that 10 min heating at 80 °C gave good results. The procedure was based on optimization data obtained by adjusting time and temperature variables. For reactions held at the highest temperature for 10 min, a temperature of 70–90 °C gave complete reaction to form a single product. Varying the reaction time from 5 to 15 min had little effect on the results. The results in Table 1 are consistent with reported conditions used in microwave triazole synthetic procedures [38,39]. An early report of a microwave method applied to triazoles employed a one-pot method at 125 °C starting with NaN_3_, an organic halide and a terminal alkyne [40]. We elected to keep the starting materials the same for each of the methods used for comparison. Therefore, a one-pot method was not employed in this paper. The reported method offers the additional advantage of microwaves of not having to process an organic azide and is “greener”, as it requires only a single stage synthesis [40].

**Figure 1:**
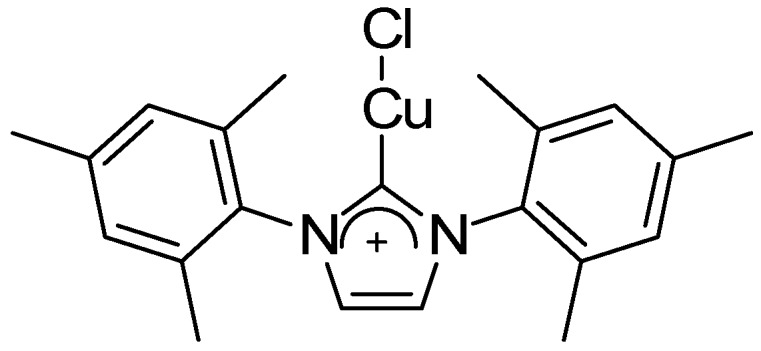
NHC-catalyst

For the solvent-free method, a conveniently prepared NHC catalyst shown in Figure 1, (1,3-bis(2,4,6-trimethylphenyl)imidazol-2-ylidene) copper(I) chloride [41], was mixed with the reactants, one of which had to be an oil, in the absence of solvent [42]. The solvent-free procedure employed a catalyst that mixed well with the oily reactants. Mixing the carbene catalyst in a liquid mixture of reactants caused an exotherm after several minutes followed by a return to room temperature after 1 h or less. NHC catalysts are known to be effective in facilitating conjugate additions [43]. Such air-stable catalysts [44,45] have been used to prepare peptidotriazoles [46]. Recently, a report appeared of an environmentally friendly fluorinated carbene catalyst employed for the preparation of triazoles in a solvent such as hexane at 40 °C [47].

The CuI/glycerol method was based on the account of Vidal and García-Álvarez [30]. A recent report used a diethylamine additive and a Schlenk line in the glycerol method for synthesis of several triazoles in good to excellent yield [48]. We chose an air environment for our experiments because the other three methods were done in air and because the yields of the earlier report using CuI in glycerol gave good yields [30].

**Scheme 1:**
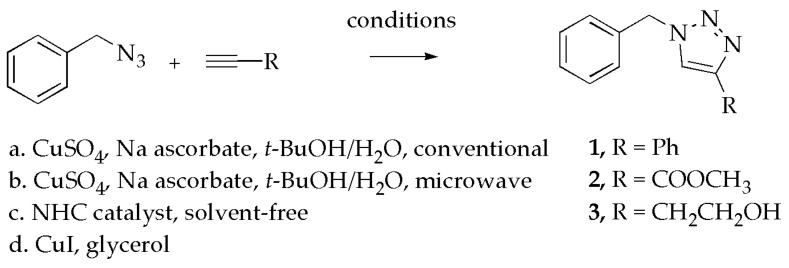
Preparation of triazoles.

The four experimental methods and products prepared are shown in Scheme 1. The triazoles synthesized were 1-benzyl-4-phenyl-1*H*-1,2,3-triazole (**1**), methyl 1-benzyl-1*H*-1,2,3-triazole-4-carboxylate (**2**), and 2-(1-benzyl-1*H*-1,2,3-triazol-4-yl)-ethanol (**3**). For the conventional method (a), heating was for 2–3 h for the non-fluorinated triazoles at 60–65 °C. In method (b), microwave heating was for 10 min at 80 °C. For the NHC method (c), the oily mixture of benzyl azide and an alkyne were mixed with the copper carbene complex catalyst under solvent-free conditions for 30–60 min. For method (d), benzyl azide was mixed with an alkyne in glycerol and the CuI catalyst for 24 h. All reactions were stirred magnetically. Isolation procedures were either a collection of solid products by filtration or isolation of oily products following extraction. A summary of results for the preparation of **1**–**3** is shown in Table 2. Yields are for the crystallized products. The low-melting **3** crystallized over time upon thorough drying.

The microwave method provided consistent results with high yields and good purity in the shortest time necessary to complete a reaction. Thin-layer chromatography (TLC) analysis of non-microwave reaction mixtures prior to working up sometimes showed the remaining starting material, whereas the microwave method gave products of high purity as judged by ^1^H-NMR spectroscopy, indicating a complete reaction. An excess of alkyne (notes 4 and 5 in Table 2) was employed for some trials of the solvent-free and CuI in glycerol methods, substantially increasing the yields. These results indicate that each method provides good yields and product purity overall, particularly if reactions achieve completion (refer to Supplemental Information for ^1^H-NMR spectra).

Scaling microwave experiments to an industrial scale has been reported [49]. We did not explore such experiments, but we scaled the four protocols for the synthesis of **1** to 20 mmol of both benzyl azide and ethynylbenzene. The microscale method afforded the highest yield (89.7%) with the highest purity. The microwave product showed a clean ^1^H-NMR spectrum, and the solvent-free product showed some trace impurities. The conventional and CuI in glycerol products contained some starting benzyl azide. These experiments were not developed further (refer to Supplemental Information for ^1^H-NMR spectra).

### 2.2. Fluorinated Triazoles

**Figure 2:**
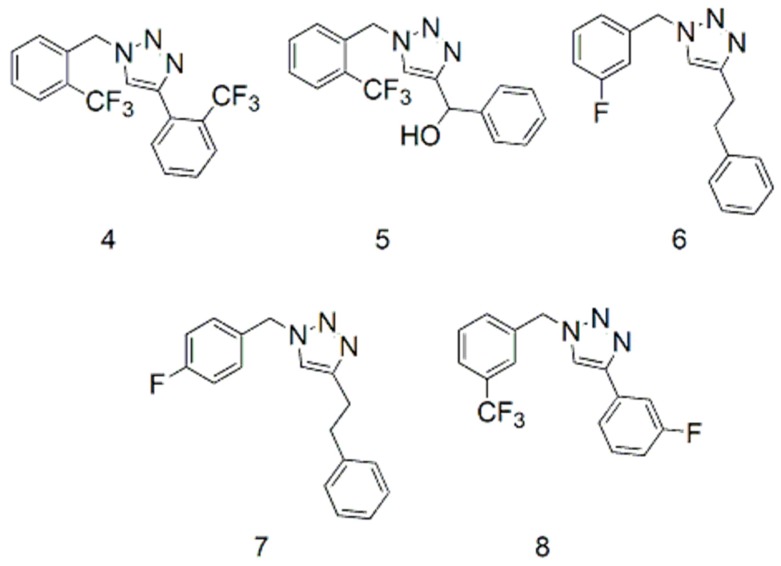
Structures of the fluorinated triazoles.

The fluorinated triazoles chosen were of interest for biological testing. Similar reaction conditions were selected for comparison of results by the four methods. The reactions consisted of fluorinated or trifluoromethylated benzyl azides and five terminal alkynes to afford 1-(2-trifluoromethylbenzyl)-4-(2-trifluoromethylphenyl)-1*H*-1,2,3-triazole (**4**), phenyl-[1-(2-trifluoromethylbenzyl)-1*H*-1,2,3-triazol-4-yl]-methanol (**5**), 1-(3-fluorobenzyl)-4-phenethyl-1*H*-1,2,3-triazole (**6**), 1-(4-fluorobenzyl)-4-phenethyl-1*H*-1,2,3-triazole (**7**), and 4-(3-fluorophenyl)-1-(3-trifluoromethylbenzyl)-1*H*-1,2,3-triazole (**8**). Structures are shown in Figure 2.

Four synthetic methods were used as in the previous set of experiments. The results are shown in Table 3. The yields and relative purity of the products were more consistent overall than for the non-fluorinated triazoles. Yields were higher for the microwave and solvent-free methods, whereas the purity was better for the microwave and conventional methods. Further purification for all products was accomplished by flash column chromatography or recrystallization from hexanes. The time needed for each method favored the microwave and solvent-free methods. On the small laboratory scale used for these experiments, each method gave a successful outcome, with the microwave method showing both higher yield and product purity.

## 3. Conclusions

Each of the four methods gave the desired triazole products. The microwave method was the quickest for the preparation of triazoles on both smaller and larger scales and gave triazoles of high purity in good yield for all trials attempted without any notable drawbacks. A one-pot procedure [40,50] could further benefit the microwave method, shortening the time needed to make the azide in situ as well as synthesizing the triazole. The solvent-free method also provided good overall conversion in a short time. All four methods have good green attributes, each having a catalyst, a green or no solvent, good atom economy, and little or no hazardous waste.

## 4. Materials and Methods

All starting materials were purchased from commercially available sources and used as obtained. All synthesized organic azides were stored at 0 °C until needed. All azides were considered as hazardous and potentially explosive. Plastic or ceramic spoons were used when weighing solid azides. All reactions were performed in a ventilated hood. Thin layer chromatography (VWR International, Radnor, PA, USA) was performed on Agela Technologies aluminum-backed silica dioxide plates and the products were observed under 254 nm UV light. Flash column and radial chromatography (T-Squared Technology, San Bruno, CA, USA) were performed with SiliCycle silica gel 60, 0.040−0.063 mm (230−400 mesh). Microwave-assisted synthesis was performed using a CEM Discover SP Microwave Synthesizer (CEM Corp., Mathews, NC, USA) set at a maximum power of 200 watts. NMR spectra (300 or 400 MHz for ^1^H, 100 MHz for ^13^C and 282 MHz for ^19^F, (Bruker Biospin Corp., Billerica, MA, USA) were measured in CDCl_3_ or DMSO-*d*_6_. Chemical shifts (δ) were given in ppm relative to the resonance of their respective residual solvent peak, CHCl_3_ (7.27 ppm, ^1^H; 77.16 ppm, the middle peak, ^13^C). Multiplicities were described using the following abbreviations: s = singlet, d = doublet, t = triplet, q = quartet, m = multiplet. FTIR experiments were performed using a Perkin Elmer Spectrum 1 instrument (PerkinElmer Corp., Waltham, MA, USA) or Fisher Scientific Nicolet iS5 instrument (Thermo Fisher Scientific Co., Waltham, MA, USA). Melting points were performed on a Stanford Research Systems Digimelt MPA160 SRS instrument (Stanford Research Systems, Sunnyvale, CA, USA). All melting points were uncorrected. All elemental analyses were performed by Atlantic Microlabs Inc., Norcross, GA, USA. High-resolution mass-spectrometry (MS) experiments were recorded using a Applied Biosystems Voyager DE-STR MALDI-TOF (ABI) instrument (JBI Scientific, Huntsville, TX, USA).

### 4.1. General Procedure for the Synthesis of Organic Azides

Metal azides are shock sensitive and thus were handled with smooth-edged, non-metallic spoons or spatulas. Purification of initially isolated products was required to remove traces of azides. Inorganic azides remaining in aqueous solutions were quenched by aqueous nitrous acid.

Into a 250 mL round-bottom flask, organic bromide (10 mmol), *tert*-butyl alcohol/water (1:1, 100 mL), and sodium azide (0.98 g, 15 mmol) were added in that order. The mixture was stirred at rt for 2–3 h. The mixture was extracted with ethyl acetate (3 × 25 mL). The organic layers were combined and washed with brine, dried over anhydrous sodium sulfate, and evaporated in vacuo to dryness. Air was gently passed over the oily product to remove traces of ethyl acetate, affording the organic azide in 80–95% yield. The azide was then stored at 0 °C and used directly without further purification.

Azidomethylbenzene [51,52]: Yellow oil. (1.19 g, 87.6%); ^1^H-NMR (CDCl_3_, 400 MHz). δ 7.39–7.29 (m, 5H), δ 4.31 (s, 2H); FTIR cm^−1^ 3031, 2089, 1597, 1453, 1252.

1-(Azidomethyl)-2-(trifluoromethyl)benzene [53]: Yellow oil. (1.92 g, 95.8% yield); ^1^H-NMR DMSO-*d*_6_, 400 MHz) δ 7.73–7.71 (m, 1H), δ 7.63–7.57 (m, 2H), δ 7.48–7.44 (m, 1H), δ 4.58 (s, 2H); FTIR cm^−1^ 2096, 1311, 1108, 1037, 765.

1-(Azidomethyl)-3-(trifluoromethyl)benzene [54]: Dark yellow oil. (1.44 g, 89.0% yield); ^1^H-NMR (CDCl_3_, 400 MHz) δ 7.92–7.90 (d, 2H), δ 7.65–7.61 (t, 1H), δ 7.52–7.48 (t, 2H), δ 4.57 (s, 2H); FTIR cm^−1^ 3062, 2096, 1691, 1596, 1448, 1213.

1-(Azidomethyl)-3-(fluoro)benzene [55]: Yellow oil. (1.586 g, 99.7% yield); ^1^H-NMR (CDCl_3_, 400 MHz) δ 7.38–7.33 (m, 1H), δ 7.10–7.08 (d, *J* = 8.2 Hz, 1H), δ 7.05–7.01 (m, 2H), δ 4.34 (s, 2H).

1-(Azidomethyl)-4-(fluoro)benzene [56]: Yellow oil. (1.586 g, 99.7% yield); ^1^H-NMR (CDCl_3_, 400 MHz) δ 7.33–7.25 (m, 2H), δ 7.12–7.05 (m, 2H), δ 4.31 (s, 2H).

#### 4.1.1. Procedure for the Conventional Synthesis of 4-Phenyl-1-(phenylmethyl)-1*H*-1,2,3-triazole (**1**). Data from One of the Runs

To a round-bottom flask or scintillation vial containing a stir bar, benzyl azide (0.533 g, 4.0 mmol), 1:1 *t*-butyl alcohol/water (10 mL), 4.0 mmol ethynylbenzene (0.409 g, 4.0 mmol), 1 M copper sulfate pentahydrate (200 μL), and sodium ascorbate (50 mg) were added. The round-bottom flask was fitted with a water-cooled reflux condenser, or the vial was capped. The solution was heated at 60 °C and monitored by TLC (2 h). The reaction mixture was cooled, and water (2 mL) was added. While stirring on ice, 10% aqueous ammonia (5 mL) was added. The reaction mixture was filtered, and the dry yellow product, mp 126.6–128.4 °C (0.634 g, 67.4%), was purified using flash column chromatography with ethyl acetate/hexanes. The purified product was a white solid with the percent recovery of 51.3%, mp 129.7–130.0 °C, 126–127 °C [57]. ^1^H-NMR (CDCl_3_, 400 MHz) δ 7.80 (m, 2H), δ 7.66 (s, 1H), δ 7.39 (m, 5H), δ 7.32 (m, 3H), δ 5.59 (s, 2H); ^13^C-NMR (CDCl_3_, 100 MHz): δ 147.69, 134.44, 130.44, 129.07, 128.72, 128.08, 127.97, 125.59, 119.39, 54.14 ppm; FTIR cm^−1^: 3141.4, 1494.1, 1045.9.

#### 4.1.2. Procedure for the Conventional Synthesis of Methyl 1-(Phenylmethyl)-1*H*-1,2,3-triazole-4-carboxylate (**2**). Data from One of the Runs

To a 100 mL round-bottom flask or scintillation vial containing a stir bar, benzyl azide (0.533 g, 4.0 mmol), *tert*-butyl alcohol/H_2_O (1:1) solution (10 mL), methyl propiolate (345 mg, 4.1 mmol), 1 M copper sulfate pentahydrate solution (200 μL), and sodium ascorbate (40 mg, 0.20 mmol) were added in that order. The round-bottom flask was fitted with a water-cooled reflux condenser, or the vial was capped. The solution was heated at 60–65 °C and monitored by TLC (3 h). Upon completion, the reaction was cooled to room temperature. A 10% ammonia solution (20 mL) was added to the mixture and stirred for 5 min. The reaction mixture was filtered, and the dry crude yellow product, mp 103.8–104.7 °C (0.726 g, 83.6%), was purified using flash column chromatography with ethyl acetate/hexanes to give a white solid with the percent recovery of 92.7%, mp 106.1–106.4 °C, 104–107 °C [58]. ^1^H-NMR (CDCl_3_, 400 MHz) δ 7.97 (d, 1H), δ 7.38 (m, 3H), δ 7.29 (m, 2H), δ 5.60 (s, 2H), δ 3.93 (s, 3H); ^13^C-NMR (CDCl_3_, 100 MHz) δ 160.99, 140.19, 135.56, 129.24, 129.08, 128.19, 127.29, 54.40, 52.20; FTIR cm^−1^: 3109.6, 3068.2, 2999.9, 2950.2, 1722.1, 1540.2, 1432.0, 1206.3, 1046.8, 1019.1, 711.5.

#### 4.1.3. Procedure for the Conventional Synthesis of 1-(Phenylmethyl)-1*H*-1,2,3-triazolyl-4-ethanol (**3**) [40] Data from One of the Runs

To a 100 mL round-bottom flask or scintillation vial containing a stir bar, benzyl azide (0.533 g, 4.0 mmol), *tert*-butyl alcohol/H_2_O (1:1) solution (10 mL), 3-butyn-1-ol (0.280 g, 4.0 mmol), 1 M copper sulfate pentahydrate solution (200 μL), and sodium ascorbate (40 mg, 0.20 mmol) were added in that order. The round-bottom flask was fitted with a water-cooled reflux condenser, or the vial was capped. The solution was heated at 60–65 °C and monitored by TLC (2 h). After completion (2 h), the reaction was cooled, and water (10 mL) was added. While stirring on ice, 10% aqueous ammonia (5 mL) was added. The reaction mixture was extracted with ethyl acetate (3 × 10 mL). The combined organic layers were washed with brine, dried over anhydrous sodium sulfate, filtered, and the solution was evaporated in vacuo to dryness to give a colorless oil, which crystallized after co-evaporating with 2-propanol and on standing in air, mp 44.9–45.9 °C. Flash chromatography with ethyl acetate/hexanes gave **3** (493 mg, 70.3%), mp 48.1–48.6 °C, 90–91 °C [40]; ^1^H-NMR (400 MHz, DMSO-*d*_6_): δ 7.91 (s, 1H), δ 7.35 (m, 5H), δ 5.54 (s, 2H), δ 4.70 (t, 1H), δ 3.62 (q, 2H), δ 2.76 (t, 2H); ^13^C-NMR (100 MHz, DMSO-*d*_6_) δ 144.70, 136.17, 128.63, 127.95, 127.81, 122.47, 60.25, 52.54, 29.07; FTIR cm^−1^: 3212.1, 3120.1, 3056.7, 2950.9, 2856.6, 1542.3, 1454.0, 1216.8, 1127.6, 1049.9, 1014.3, 694.1.

#### 4.1.4. Procedure for the Microwave Preparation of Triazoles **1**–**3**

To a microwave vessel (35 mL) containing a stir bar, benzyl azide (0.533 g, 4.0 mmol), 1:1 *t*-butyl alcohol/water (10 mL), a terminal ethyne (4.0 mmol), 1 M copper sulfate pentahydrate (200 μL), and sodium ascorbate (50 mg) were added. The capped vessel was heated in the microwave instrument using the following method. The temperature was ramped over 1 min to 80 °C and held for 10 min as the pressure increased but remained below 2 atm. The vial was vented and cooled to about 50 °C. The cap was removed from the vial, and ice water (2 mL) was added followed by 10% aqueous ammonia (5 mL), and the solution was stirred for 5 min followed by extraction with ethyl acetate/brine (3 × 10 mL). The crystallized products **1** and **2** were filtered and air-dried, whereas extraction with ethyl acetate (3 × 10 mL) and drying the organic layer with sodium sulfate and evaporation of the solvent gave **3** as an oil, which crystallized upon placing it in vacuo. Alternatively, the oil crystallized upon standing in air overnight.

#### 4.1.5. Procedure for the NHC Preparation of Triazoles **1**–**3**

Benzyl azide (0.533 g, 4.0 mmol) was mixed with a terminal alkyne (2 mmol, 0.240 g). The NHC catalyst (40 mg, 0.1 mmol) was added. During 30 min, a mild exotherm was noted, and the mixture was dissolved in ethyl acetate, washed with brine (3 × 10 mL), and the organic layer was dried with anhydrous sodium sulfate. The solvent was removed to yield solid **1** or **2**. An oil was obtained with **3**, which crystallized upon standing in air.

#### 4.1.6. Procedure for the CuI/Glycerol Preparation of Triazoles **1**–**3**

Benzyl azide (0.533 g, 4 mmol) was mixed with a terminal alkyne (4 mmol) in glycerol (4 mL). CuI (8 mg) was then added, and the mixture was stirred for about 24 h. Water (2 mL) was added. While stirring on ice, 10% aqueous ammonia (5 mL) was added, followed by extraction with ethyl acetate (3 × 10 mL), drying the organic layer with sodium sulfate, and evaporation of the solvent to give solid **1** or **2**. An oil was obtained with **3**, which crystallized upon standing in air.

#### 4.1.7. Procedures for the 20 mmol Versions of the Preparation of **1**

Each of the four methods included reactants at 5× the amounts used for the 4 mmol experiments, and, where relevant, 1 M copper sulfate pentahydrate (500 μL), sodium ascorbate (100 mg), 1:1 *t*-butyl alcohol/water (17 mL). The microwave procedure was held at 90 °C. The time required for solidification in the NHC procedure was 90 min. The time allowed for the conventional heating procedure was 22 h. Yields and melting points for the microwave method were 89.7%, mp 128.4–129.5 °C; for the conventional heating method: 77.6%, mp 121.5–123.4 °C (70% conversion by ^1^H-NMR); for the NHC method: 63.3%, mp 123.2–125.8 °C; and for the CuI/glycerol method: 42.0%, mp 109.8–113.2 °C (50% conversion by ^1^H-NMR) (refer to the Supplemental Information).

#### 4.1.8. Procedures for the Preparation of 4-(3-Fluorophenyl)-1-(3-trifluoromethylbenzyl)-1*H*-1,2,3-triazole (**8**) (Similar Procedures were Used to Prepare **4**–**7**)

Conventional Method

In a 100 mL round-bottom flask with a reflux condenser (or a 20 mL scintillation vial), 1-(azidomethyl)-3-(trifluoromethyl)benzene (0.422 g, 2.1 mmol) and 1-ethynyl-3-fluorobenzene (0.240 g, 2.0 mmol) in 1:1 *tert*-butyl alcohol/water (10 mL) were added. Sodium ascorbate (59 mg), 1 M aq. copper (II) sulfate pentahydrate (100 μL), and a stir bar were added. The scintillation vial was capped. The mixture was stirred for 24 h at room temperature (**4** and **5**) or heated in a water bath at 60 °C for 3 h (**6**–**8**) and allowed to cool. Ice water (30 mL) was added, followed by 10% aqueous ammonia (6 mL), and the solution was stirred for 10 min. The blue mixture was either filtered to give an off-white solid or extracted with ethyl acetate/brine (3 × 20 mL). The organic layer was dried with anhydrous sodium sulfate, and the solvent was removed in vacuo. The product **8** was recrystallized in hexanes, mp: 61.8–62.2 °C; ^1^H-NMR (CDCl_3_, 400 MHz) δ 7.73 (s, 1H), δ 7.56 (m, 6H), δ 7.37 (q, 1H), δ 7. 02 (t, 1H) δ 5.65 (s, 2H); ^13^C-NMR (CDCl_3_, 100 MHz) δ 164.9, 161.7, 147.5, 135.6, 132.6, 132.0, 131.5, 130.6, 130.4, 129.9, 125.9, 125.5, 124.8, 121.9, 121.3, 120.0, 115.4, 115.0, 112.9, 112.6, 53.8; ^19^F-NMR (CDCl_3_, 282 MHz) δ −62.6, −112.5; FTIR cm^−1^: 3100, 1589, 1450, 1325, 1114, 1075, 864, 789. Anal. Calcd. for C_16_H_11_N_3_F_4_ %: C, 59.81; H, 3.45; N, 13.08; F, 23.65. Found: C, 59.88; H, 3.41; N, 13.81; F, 23.71.

Microwave Method

1-(Azidomethyl)-3-(trifluoromethyl)benzene (0.201 g, 1 mmol) was mixed with 1-ethynyl-3-fluorobenzene (0.120 g, 1 mmol) in 1:1 tert-butyl alcohol/water (3 mL). Sodium ascorbate (20 mg) and 1 M aq. copper (II) sulfate pentahydrate (50 μL) were added, and the mixture was heated in the microwave oven by ramping over 1 min to 100 °C and held for 2 min as the pressure rose and held below 2 atm. The system then vented the vial and cooled the mixture to about 50 °C. The cap was removed from the vial. Ice water (10 mL) was added followed by 10% aqueous ammonia (2 mL), and the solution was stirred for 5 min followed by extraction with ethyl acetate/brine (3 × 10 mL). The organic layer was dried with anhydrous sodium sulfate, and the solvent was removed in vacuo. The product **8** was recrystallized from methanol/water.

NHC Method

1-(Azidomethyl)-3-(trifluoromethyl)benzene (0.422 g, 2.1 mmol) was mixed with 1-ethynyl-3-fluorobenzene (0.240 g, 2 mmol). The NHC catalyst was added (0.10 mmol, 41 mg). During 30 min, a mild exotherm was noted. The mixture was dissolved in ethyl acetate, washed with brine (3 × 10 mL), and the organic layer was dried with anhydrous sodium sulfate. The solvent was removed to yield solid **8.** An oil was obtained with **3**, which crystallized upon standing in air.

CuI/Glycerol Method

1-(Azidomethyl)-3-(trifluoromethyl)benzene (0.201 g, 1 mmol) was mixed with 1-ethynyl-3-fluorobenzene (0.120 g, 1.0 mmol) in glycerol (1 g). CuI (2 mg) was added, and the mixture was stirred for 24 h. Water (5 mL) was added, and the product **8** appeared as a white precipitate, which was collected using vacuum filtration.

1-(2-Trifluoromethylbenzyl)-4-(2-trifluoromethylphenyl)-1*H*-1,2,3-triazole (**4**): mp: 72.2–72.7 °C; ^1^H-NMR (CDCl_3_, 400 MHz); δ 7.98 (d, *J* = 8 Hz, 1H), 7.74 (m, 3H), 7.64 (t, *J* = 7 Hz, 1H), 7.55 (t, *J* = 7 Hz, 1H), 7.48 (q, *J* = 7 Hz, 2H), 7.18 (d, *J* = 7 Hz, 1H), 5.83 (s, 2H); ^13^C-NMR (CDCl_3_, 100 MHz) δ 144.9, 133.1, 132.8, 132.0, 131,7, 129.8, 129.3, 128.8, 128.4, 128.2, 127.8, 127.3, 126.3, 126.1, 123.5, 122.3, 50.3; ^19^F-NMR (CDCl_3_, 282 MHz) δ −58.8, −59.4; FTIR cm^−1^: 1340.8, 1102.5, 1085.0, 1058.3,1035.2, 768.9. Anal. Calcd. for C_17_H_11_N_3_F_6_ %: C, 54.99; H, 2.99; N, 11.32; F, 30.70. Found: C, 54.71; H, 3.06; N, 11.08; F, 30.73; *m/z*: 372 ([M + H]^+^).

Phenyl-[1-(2-trifluoromethylbenzyl)-1*H*-1,2,3-triazol-4-yl]-methanol (**5**): mp: 95.8–96.3 °C; ^1^H-NMR (CDCl_3_, 400 MHz) δ 7.71 (d, 1H), δ 7.52 (t, 1H), δ 7.44 (m, 3H), δ 7.33 (m, 3H), δ 7.17 (d, 1H), δ 6.03 (s, 1H), δ 5.69 (s, 2H); ^13^C-NMR (CDCl_3_, 300 MHz) δ 141.9, 133.2, 132.8, 130.1, 128.8, 128.6, 128.2, 127.7, 126.4, 126.3, 126.1, 125.9, 122.4, 121.7, 69.3, 50.2; ^19^F-NMR (CDCl_3_, 282 MHz) δ −59.2; FTIR cm^−1^: 3212.8, 1449.6, 1312.6, 1120.0, 1036.9, 772.4, 693.9. Anal. Calcd. for C_17_H_14_N_3_F_3_O %: C, 61.26; H, 4.23; N, 12.61; F, 17.10. Found: C, 61.24; H, 4.24; N, 12.57; F, 17.05.

1-(3-Fluorobenzyl)-4-phenethyl-1*H*-1,2,3-triazole (**6**): mp: 77.0–77.4 °C; ^1^H-NMR (CDCl_3_, 400 MHz) δ 7.33 (t, 1H), δ 7.25 (t, 2H), δ 7.16 (m, 3H), δ 7.03 (m, 2H), δ 6.97 (d, 1H), δ 6.87 (d, 1H) δ 5.46 (s, 2H), δ 3.01 (o, 4H); ^13^C-NMR (CDCl_3_, 300 MHz) δ 164.8, 161.5, 141.1, 137.5, 137.4, 130.8, 130.6, 128.6, 128.4, 126.1, 123.4, 121.2, 115.7, 115.5, 115.0, 114.6, 53.4, 35.7, 27.5; ^19^F-NMR (CDCl_3_, 282 MHz) δ −111.7; FTIR cm^−1^: 3108.8, 3059.8, 3024.7, 2932.9, 1590.6, 1450.4, 1245.2, 1054.9, 710.3, 690.0. Anal. Calcd. for C_17_H_16_N_3_F %: C, 72.58; H, 5.73; N, 14.94; F, 6.75. Found: C, 72.59; H, 5.85; N, 14.97; F, 6.67.

1-(4-Fluorobenzyl)-4-phenethyl-1*H*-1,2,3-triazole (**7**): mp: 96.1–96.3 °C; ^1^H-NMR (CDCl_3_, 400 MHz) δ 7.23 (m, 2H), δ 7.17 (m, 3H), δ 7.13 (m, 2H), δ 7.03 (m, 3H), δ 5.43 (s, 2H), δ 3.01 (o, 4H); ^13^C-NMR (CDCl_3_, 300 MHz) δ 164.4, 161.1, 147.9, 141.1, 130.8, 129.8, 128.5, 126.1, 120.8, 116.2, 115.9, 53.0, 35.5, 27.5; ^19^F-NMR (CDCl_3_, 282 MHz) δ −113.1; FTIR cm^−1^: 3110.4, 3056.9, 2949.8, 2926.2, 2859.4, 1508.7, 1219.7, 1075.4, 776.8, 699.1, 530.4. Anal. Calcd. for C_17_H_16_N_3_F %: C, 72.58; H, 5.73; N, 14.94; F, 6.75. Found: C, 72.44; H, 5.83; N, 14.81; F, 6.87.

## Figures and Tables

**Table 1:** Optimization of microwave conditions using benzyl azide and ethynylbenzene, CuSO_4_, and sodium ascorbate in *t*-BuOH/H_2_O.

Experiment	Maximum Temperature °C	% Yield (Crude)	% Yield (Pure)
1	60	72.1	53.4
2	70	95.0	80.3
3	80	100.0	89.0
4	90	96.2	80.8
5	100	96.2	68.2
6	110	89.4	68.5
7	120	92.6	78.9

**Table 2:** Synthesis of triazoles by four sets of conditions (highest two trials per entry).

**Method**	**a. Conventional**			**b. Microwave**		
**Compound**	**Yield %**	**Time (h)**	**Mp °C ^1^**	**Yield %**	**Time (h)**	**Mp °C ^1^**
**1**	82.9	2.0	128.0–129.5	91.8	0.25	128.7–130.3
**2**	80.9	3.0	105.0–105.7	88.0	0.25	106.1–106.4
**3**	71.3 ^2^	2.0	40.0–40.9	92.1 ^2^	0.25	44.9–45.5
Avg.	78.4	2.3		90.6	0.25	
**Method**	**c. Solvent-Free**			**d. CuI in Glycerol**		
**Compound**	**Yield %**	**Time (h)**	**Mp °C ^1^**	**Yield %**	**Time (h)**	**Mp °C ^1^**
**1**	93.7	1.0	124.4–126.9	62.2 ^4^	24	126.6–128.4
**2**	67.9	0.5	101.8–103.9	96.9	24	103.8–104.7
**3**	68.4 ^2^	1.0	42.4–44.0	52.0	24	oil
Avg.	76.7 ^3^	0.8		70.6	24	

^1^ Melting point data are for experiments with excess alkyne. ^2^ Yield of solid after drying under vacuum. ^3^ The average yield was 91.5% based on benzyl azide when a 50% excess of alkyne was used. ^4^ The yield was 96.0% when a 50% excess of ethynylbenzene was used.

**Table 3:** Fluorinated triazoles yield and melting range ^1^.

Method	Conventional (3 h)		Microwave (5 min)		Solvent-Free (0.5 h)		CuI/Glycerol (1 day)	
Compound	Yield %	Mp, °C	Yield %	Mp, °C	Yield %	Mp, °C	Yield %	Mp, °C
**4**	76.3	72.2–72.7	96.9	71.4–72.0	96.4	68.5–70.9		
**5**	96.1	93.2–94.4	92.9	92.8–94.4	100.0	92.9–94.2	84.0	88.1–92.5
**6**	74.7	76.7–77.3	78.2	76.7–77.3	94.5	76.3–77.1	67.9	74.8–75.8
**7**	91.9	94.2–95.5	87.8	94.2–95.6	71.5	94.8–95.6	85.6	93.9–94.5
**8**	85.7	62.5–63.2	96.7	62.6–63.2	88.9	59.4–63.4	83.7	61.1–61.7
Avg.	84.9		90.5		90.3		80.3	

^1^ Yields prior to recrystallization are shown for comparison. Melting points are 0.5–3 °C below those of the recrystallized or chromatographed products.

## Supplementary Materials

NMR and FTIR spectra of compounds prepared are available in the Supplementary Material.
