# Supplementary material for: Green Methodologies for Copper(I)-Catalyzed Azide-Alkyne Cycloadditions: A Comparative Study
Source: Molecules. 2019 Mar 10;24(5):973. doi: 10.3390/molecules24050973 (PMC6429464; doi:10.3390/molecules24050973)
Supplement: Supplementary file 1 [file molecules-24-00973-s001.pdf]

Supplementary Material

Advantages of Green Methodologies-Microwave and Solvent-free

Marissa N. Trujillo, Clayton J. Hull-Crew, Andrew D. Outlaw, Kevin A. Stewart, Loren J. Taylor, Laura F. George, Allison Duensing, Breanna M. Tracey and Allen M. Schoffstall*

Department of Chemistry and Biochemistry, University of Colorado Colorado Springs (UCCS), Colorado Springs CO 80918, United States

Table of Contents

Spectra of Compounds **1-8**

Compound **1:** 1-Benzyl-4-phenyl-1*H*-1,2,3-triazole S2-S4

Compound **2:** Methyl 1-benzyl-1*H*-1,2,3-triazole-4-carboxylate S5-S7

Compound **3:** 2-(1-Benzyl-1*H*-1,2,3-triazol-4-yl)-ethanol S8-S10

Compound **4:** 1-(2-Trifluoromethylbenzyl)-4-(2-trifluoromethylphenyl)-1*H*-1,2,3-triazole S11-S18

Compound **5:** Phenyl-[1-(2-trifluoromethylbenzyl)-1*H*-1,2,3-triazol-4-yl]-methanol S19-S24

Compound **6:** 1-(3-Fluorobenzyl)-4-phenethyl-1*H*-1,2,3-triazole S25-S29

Compound **7:** 1-(4- Fluorobenzyl)-4-phenethyl-1*H*-1,2,3-triazole S30-S34

Compound **8:** 4-(3-Fluorophenyl)-1-(3-trifluoromethylbenzyl)-1*H*-1,2,3-triazole S35-S39

Spectra of Crude Compounds **1-3**

Spectra from the scale up experiments S40-S43

Spectra from equimolar experiments S44-S45

Spectra from experiments with 50% excess alkyne S46-S49

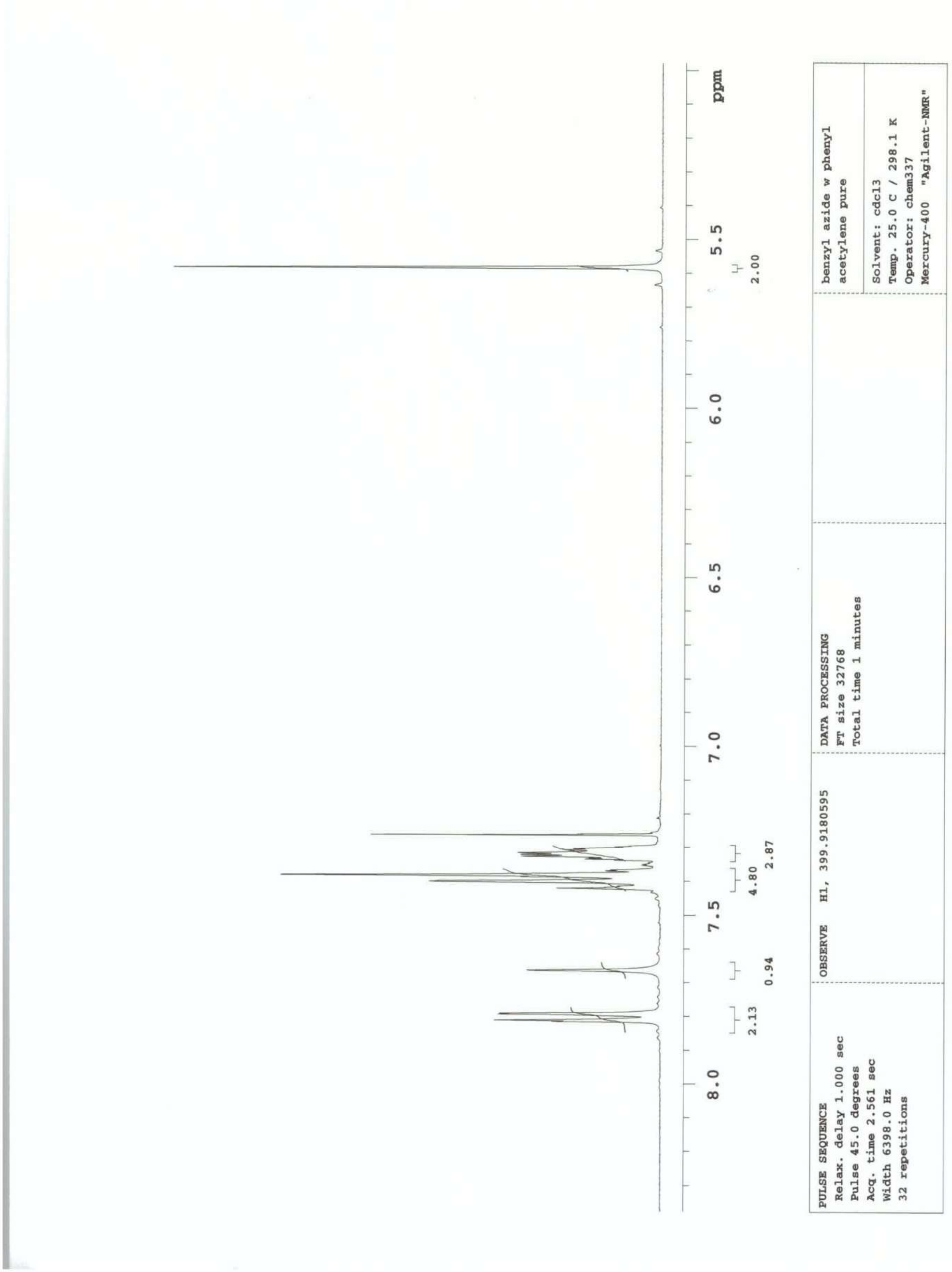

^{1}H NMR spectrum of **1**

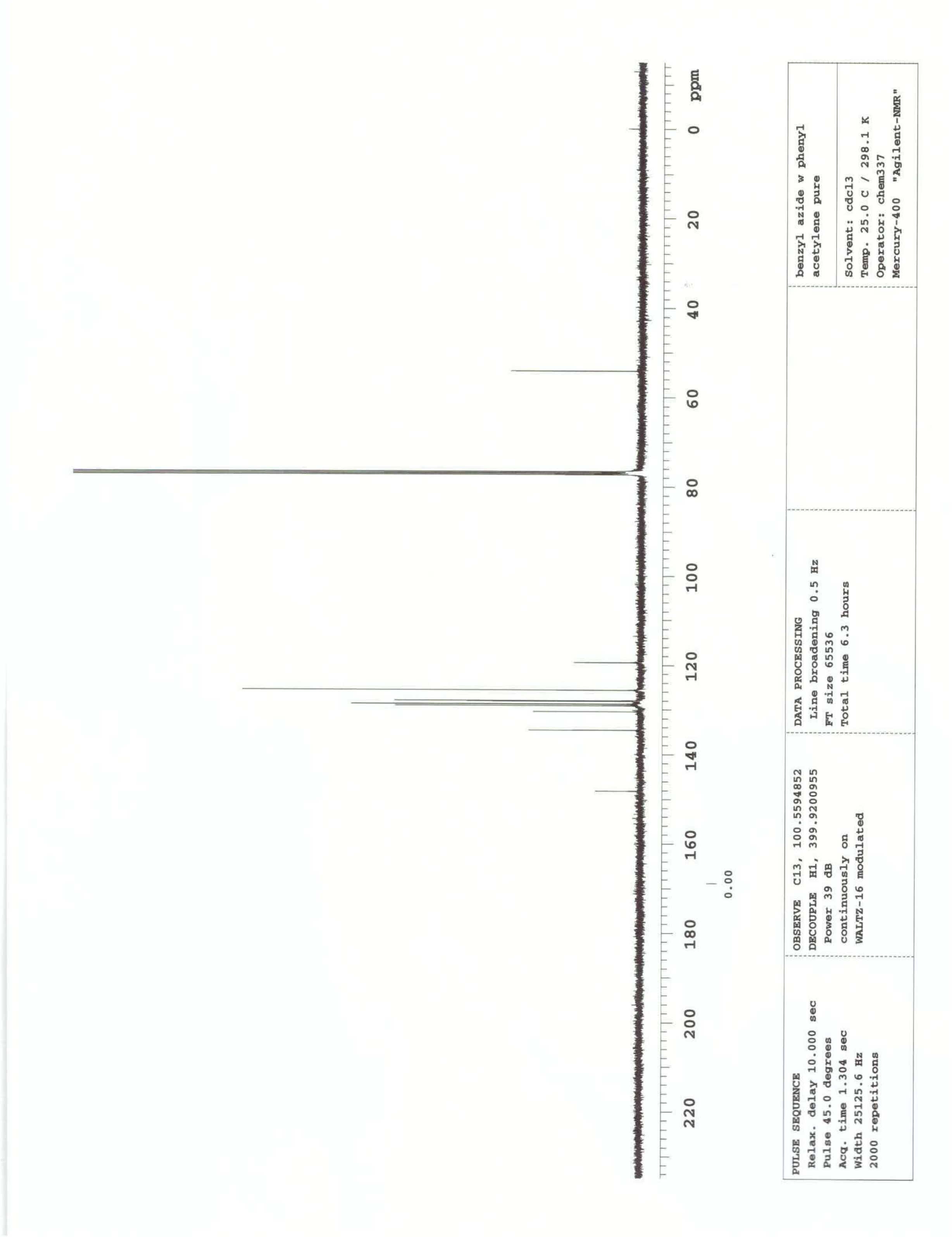

^{13}C NMR spectrum of **1**

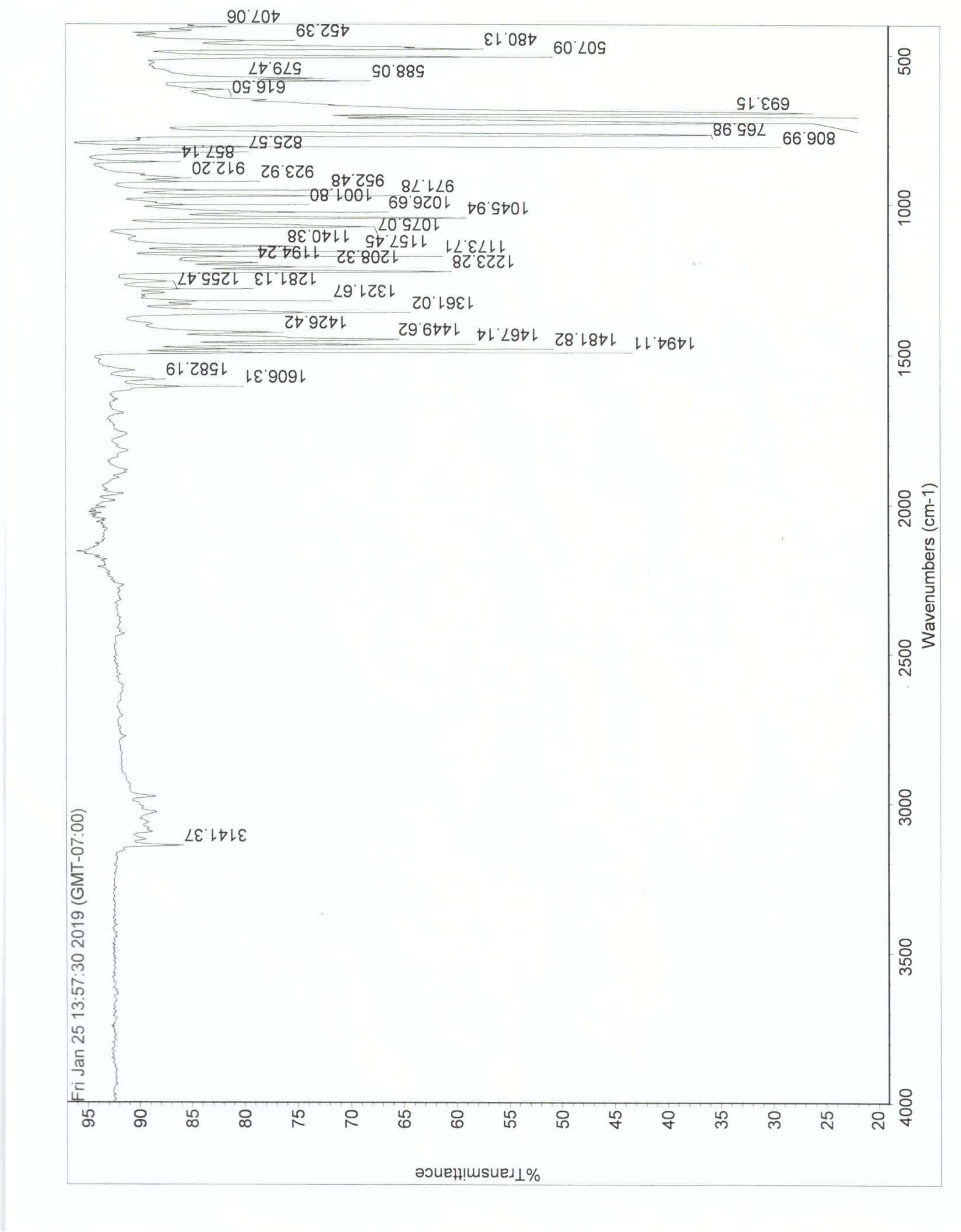

FTIR spectrum of **1**

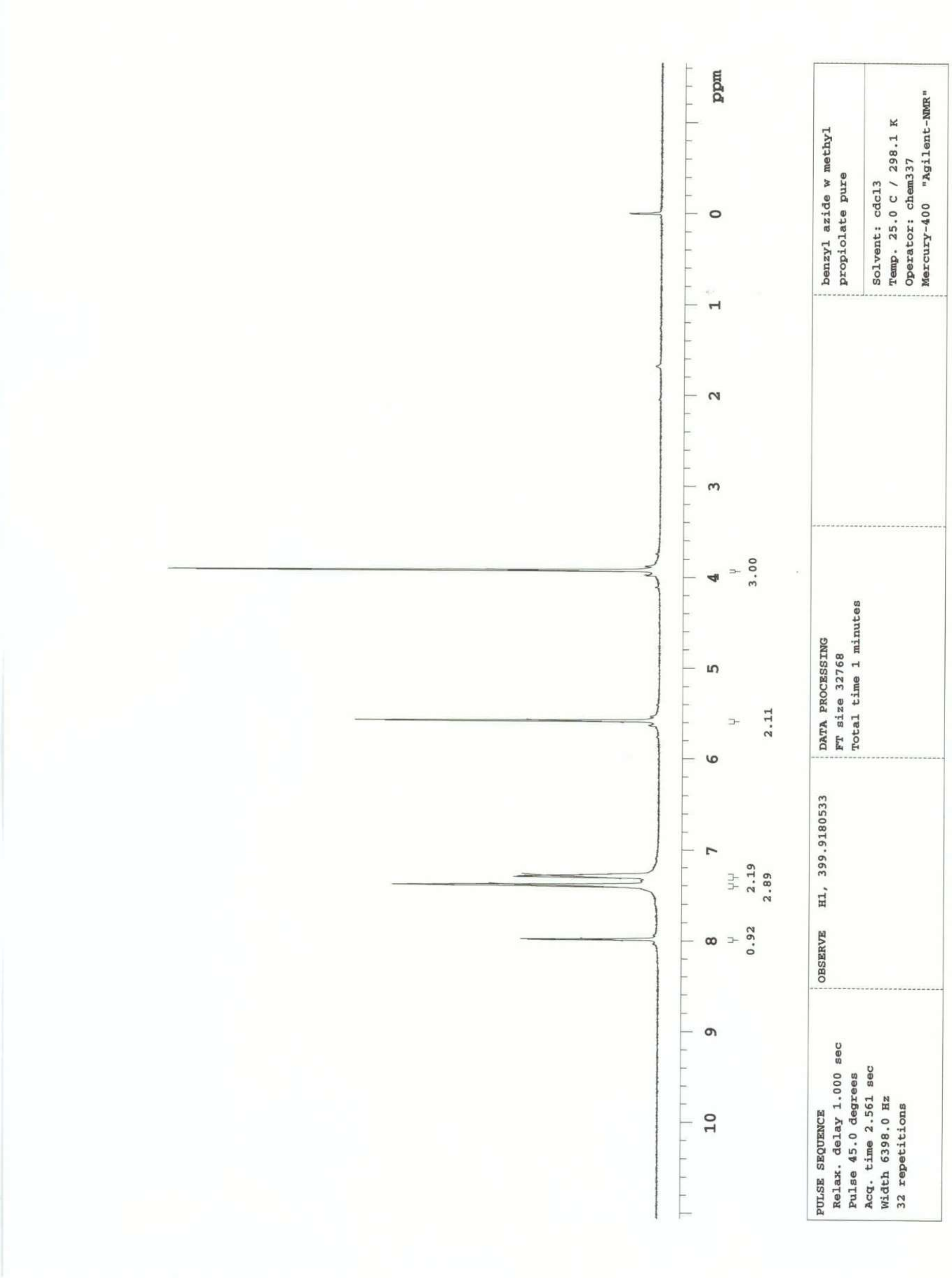

^{1}H NMR spectrum of **2**

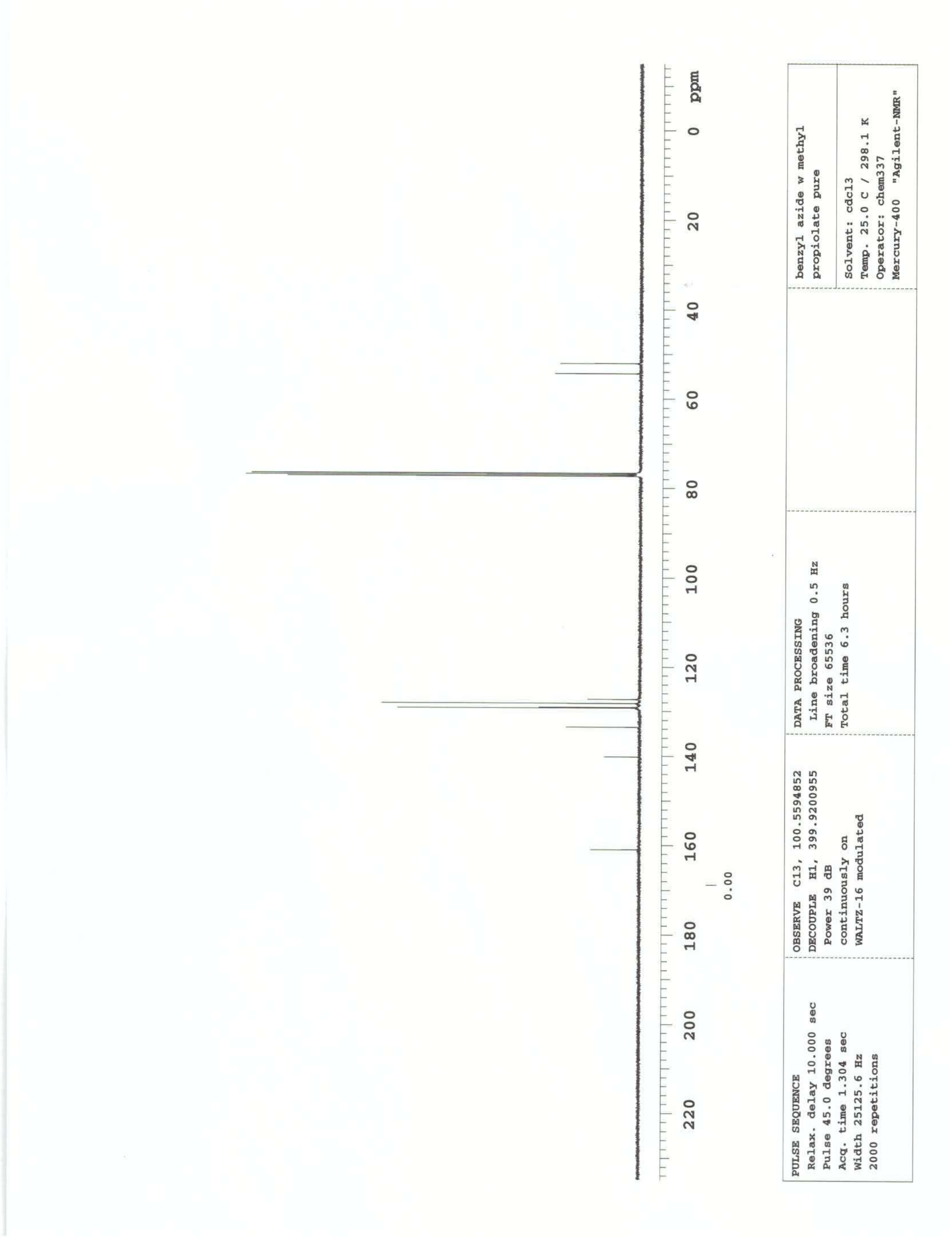

^{13}C NMR spectrum of **2**

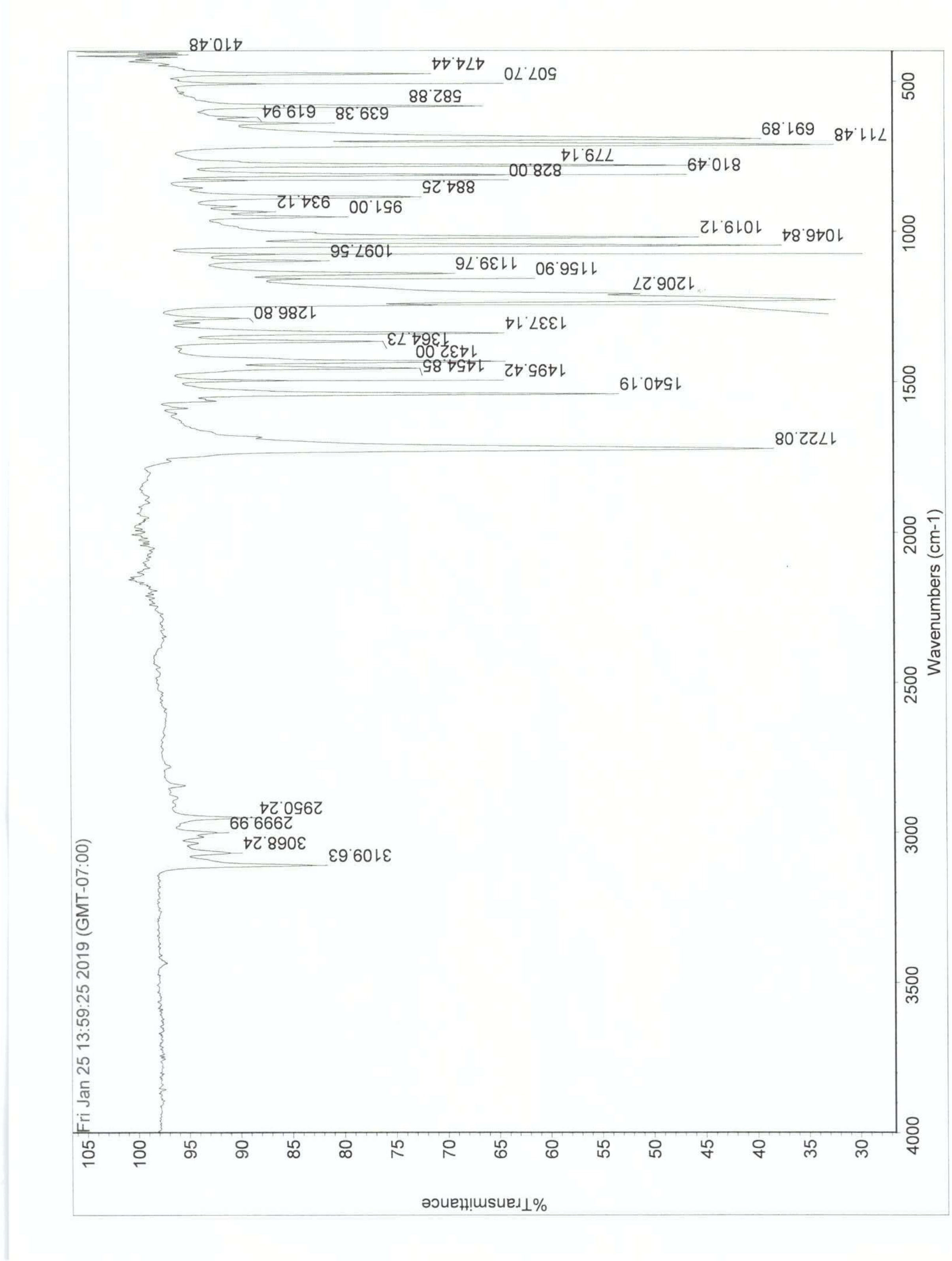

FTIR spectrum of **2**

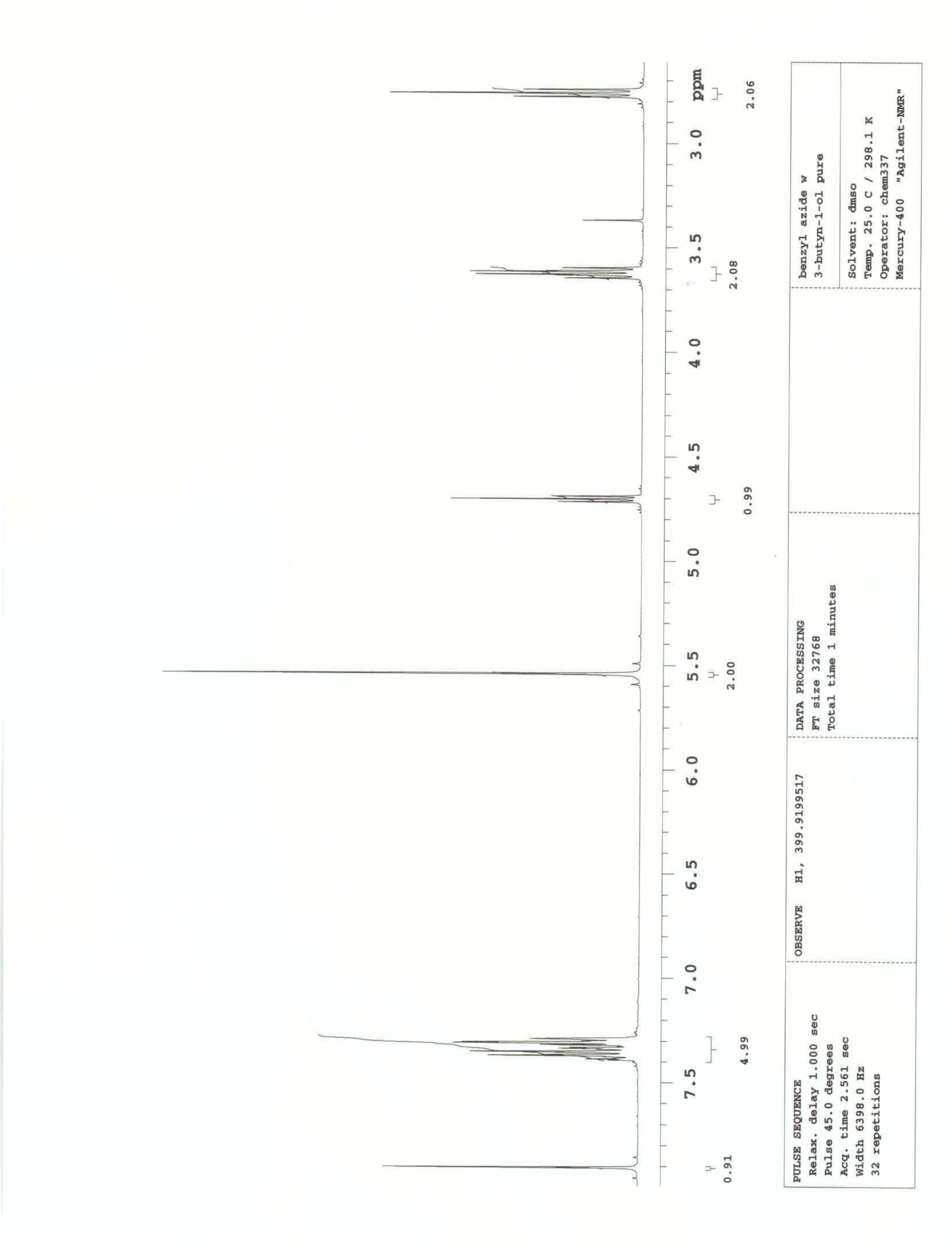

^{1}H NMR spectrum of **3**

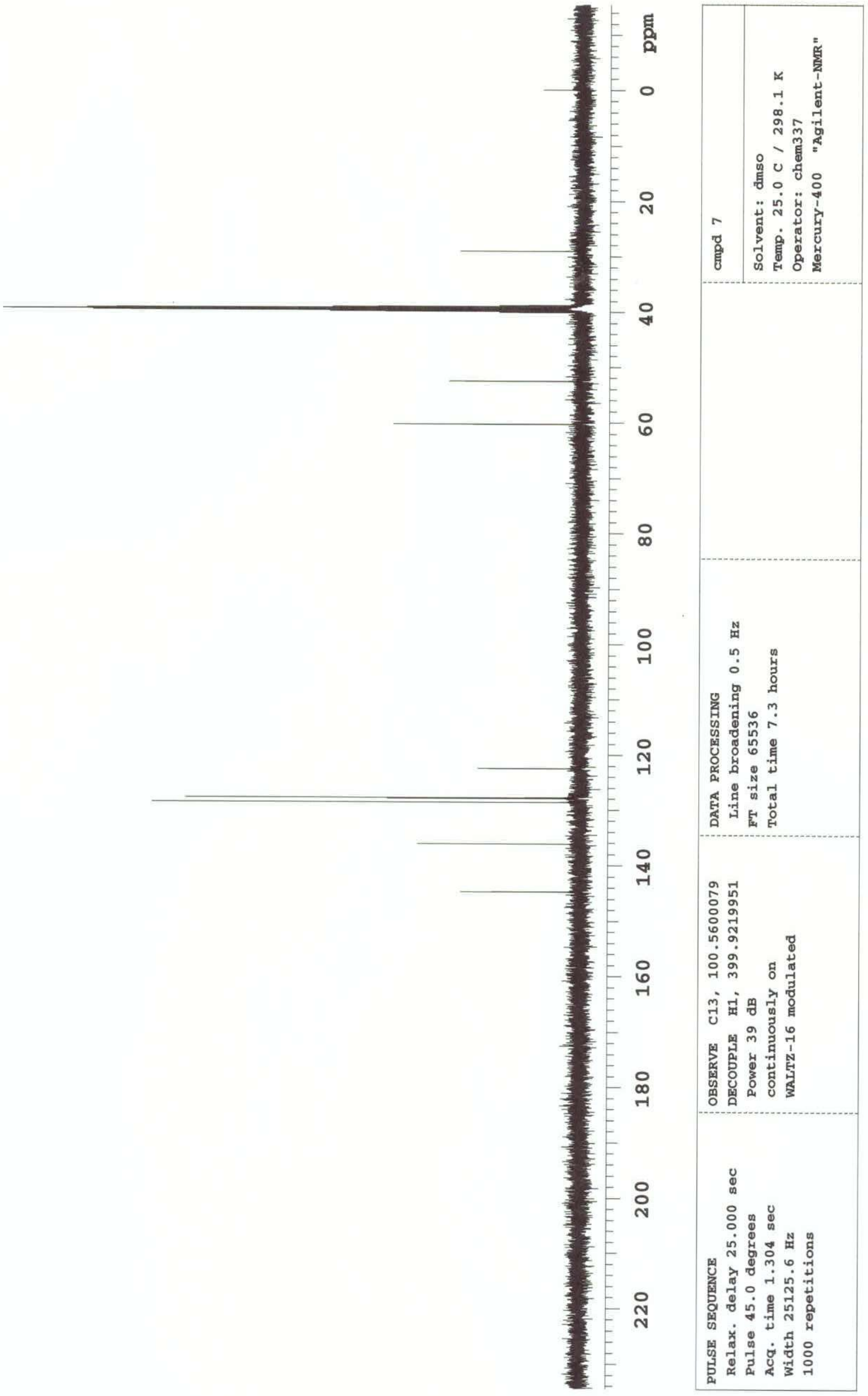

^{13}C NMR spectrum of **3**

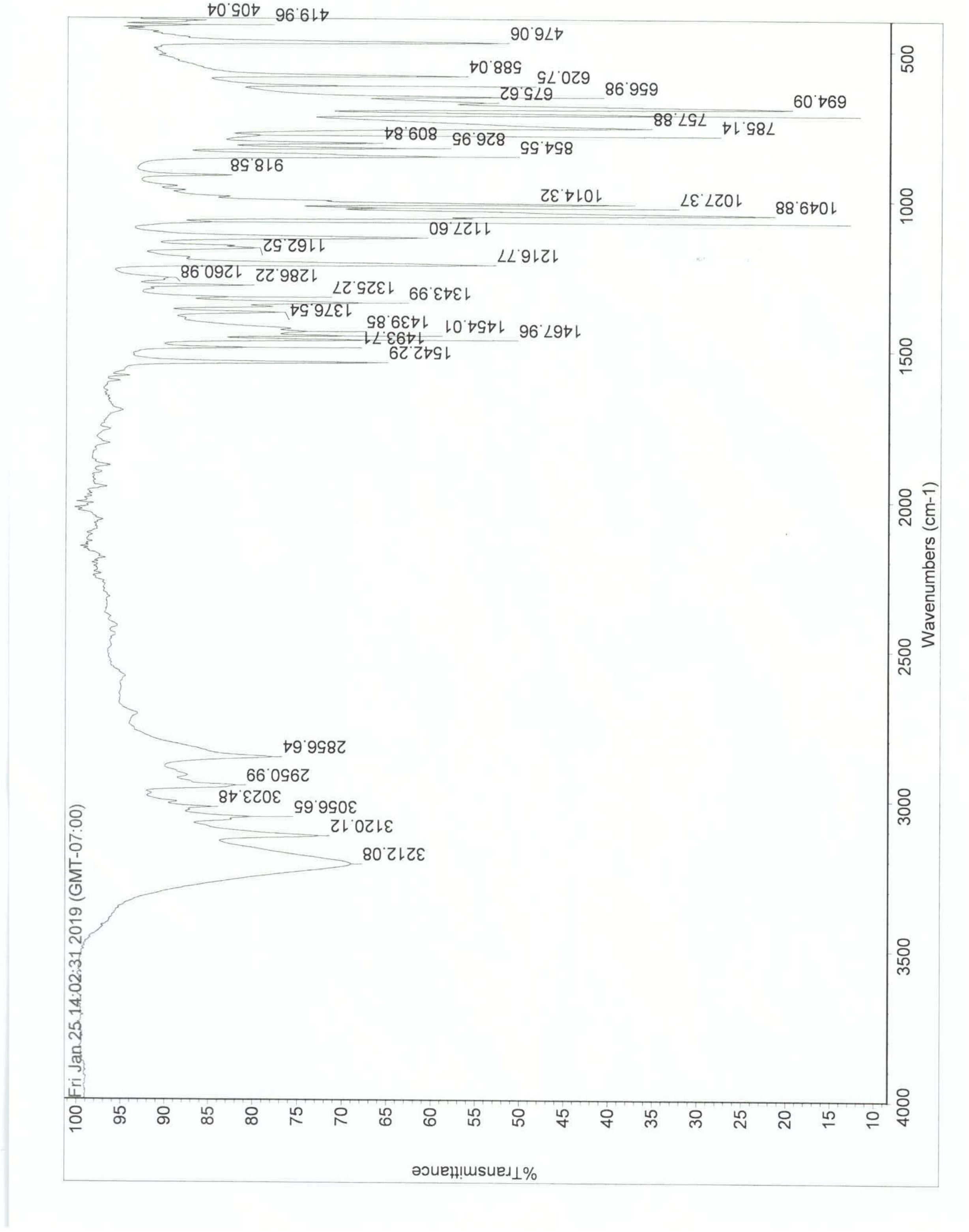

FTIR spectrum of **3**

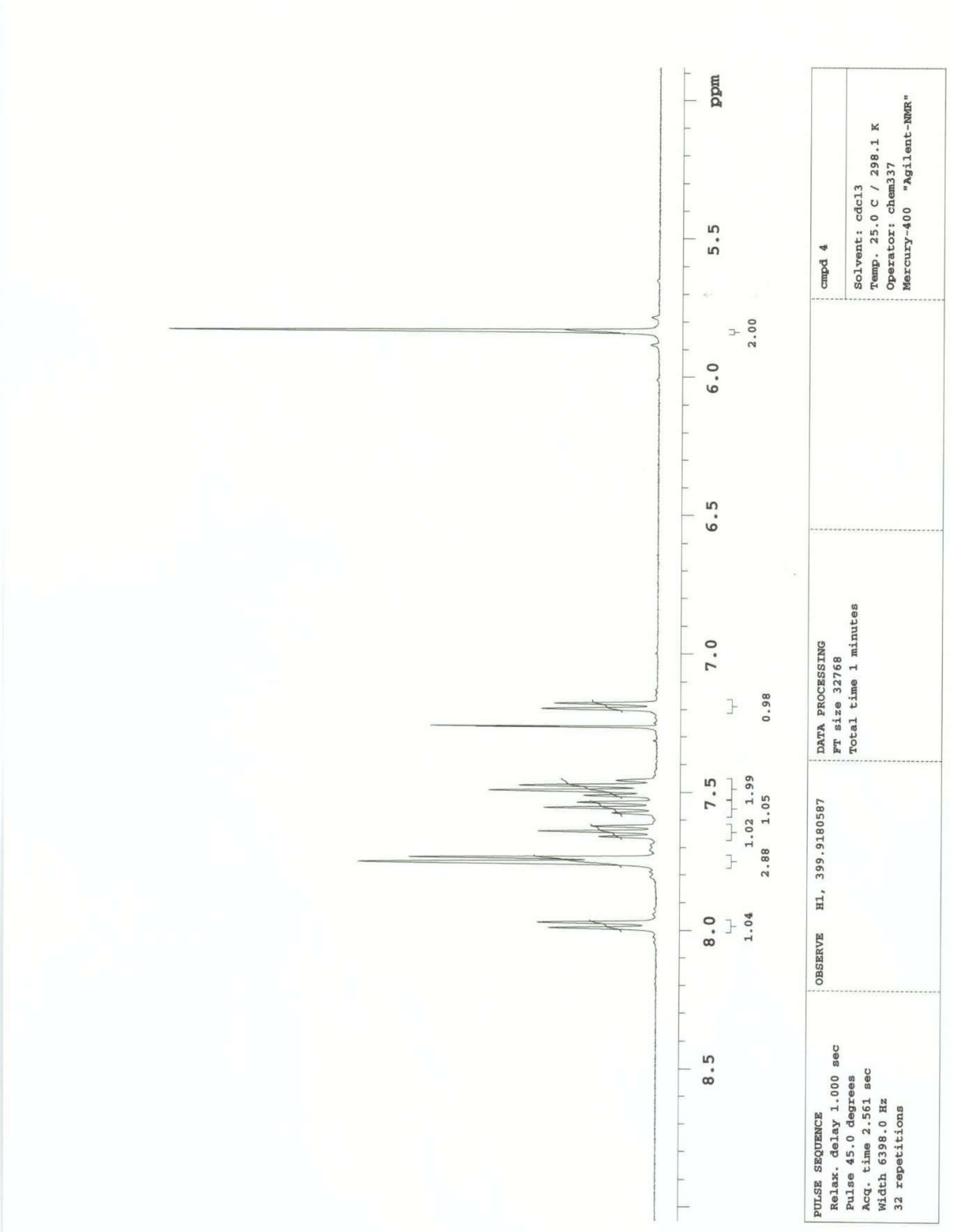

^{1}H NMR spectrum of **4**

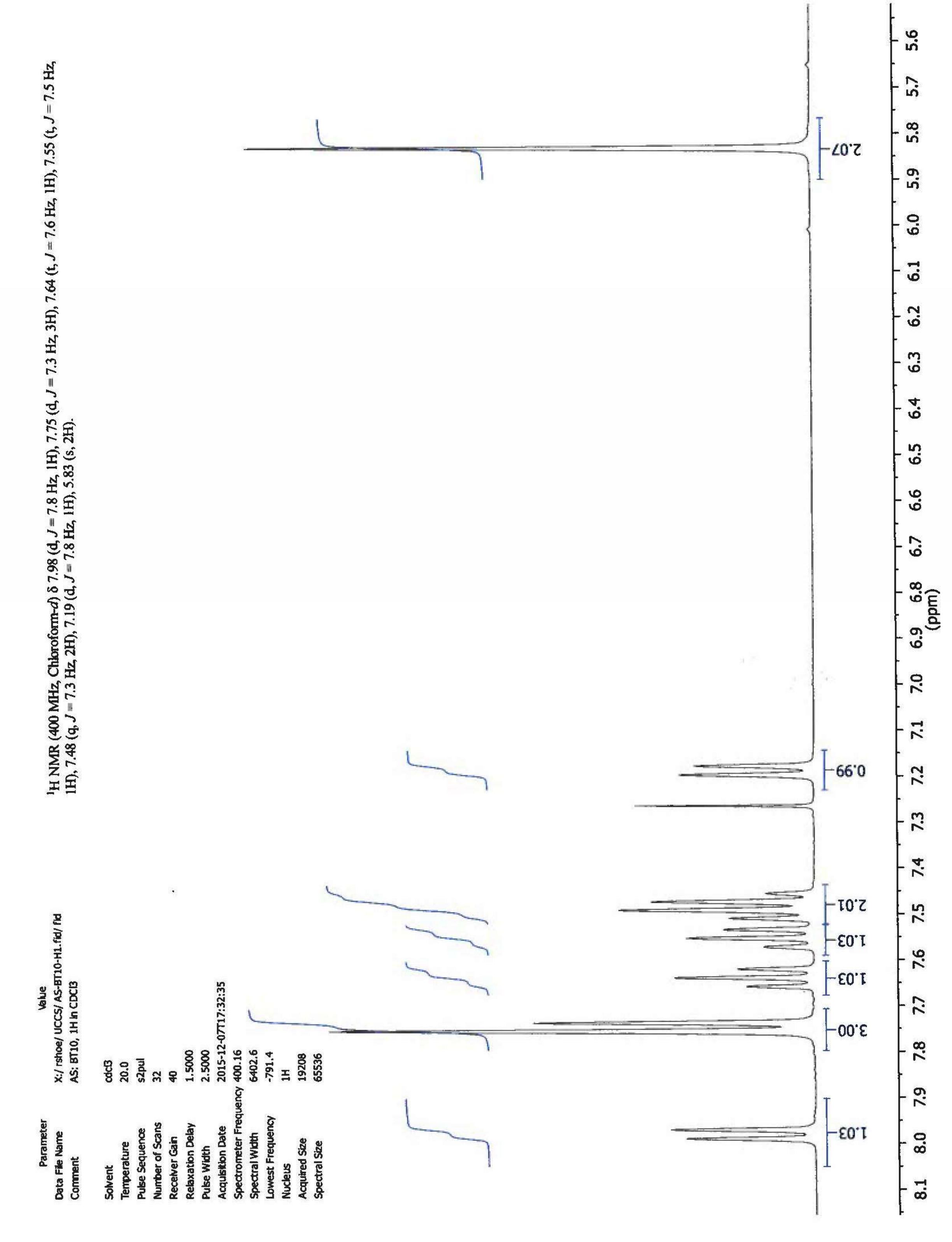

^{1}H NMR spectrum (expanded) of **4**

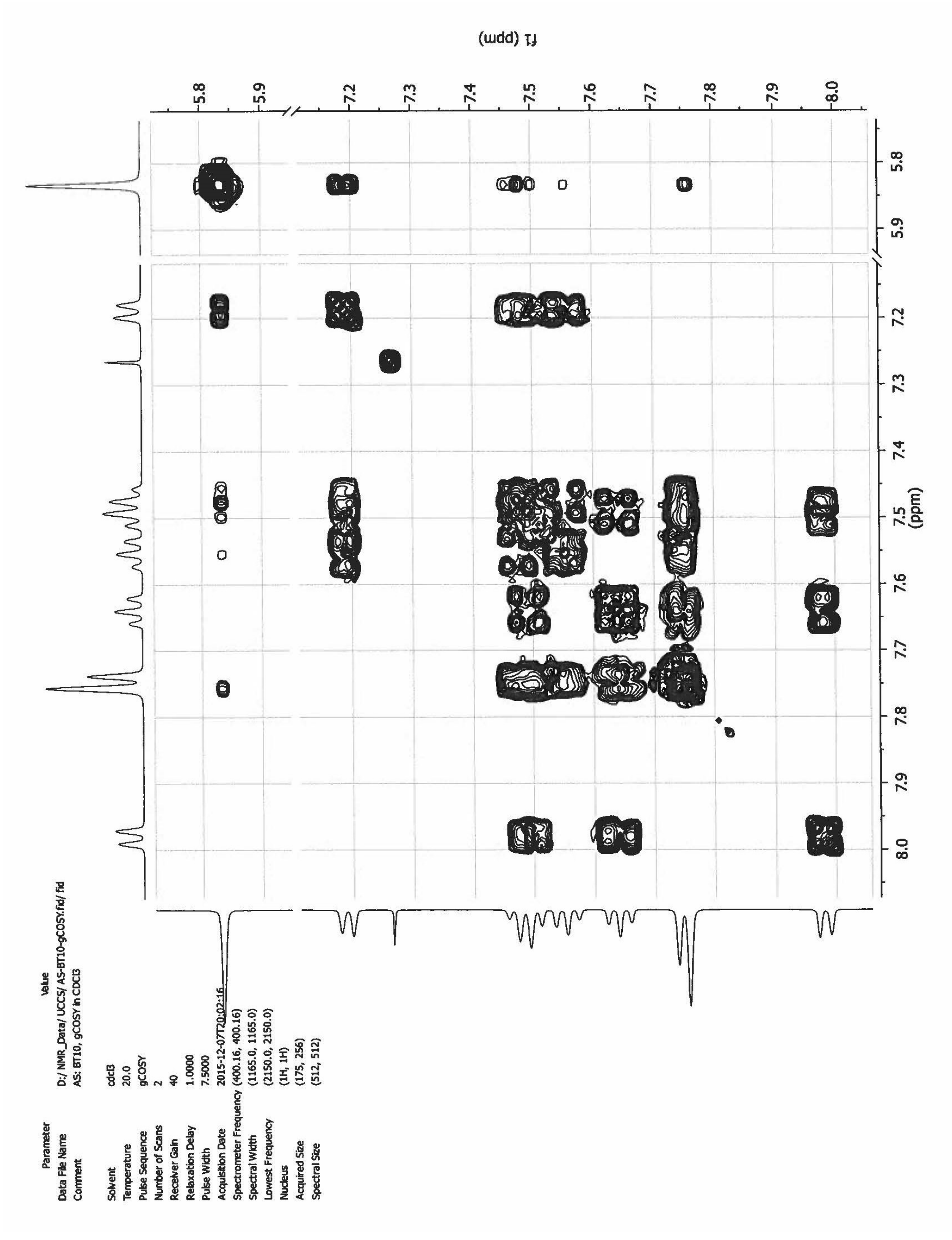

COSY spectrum of **4**

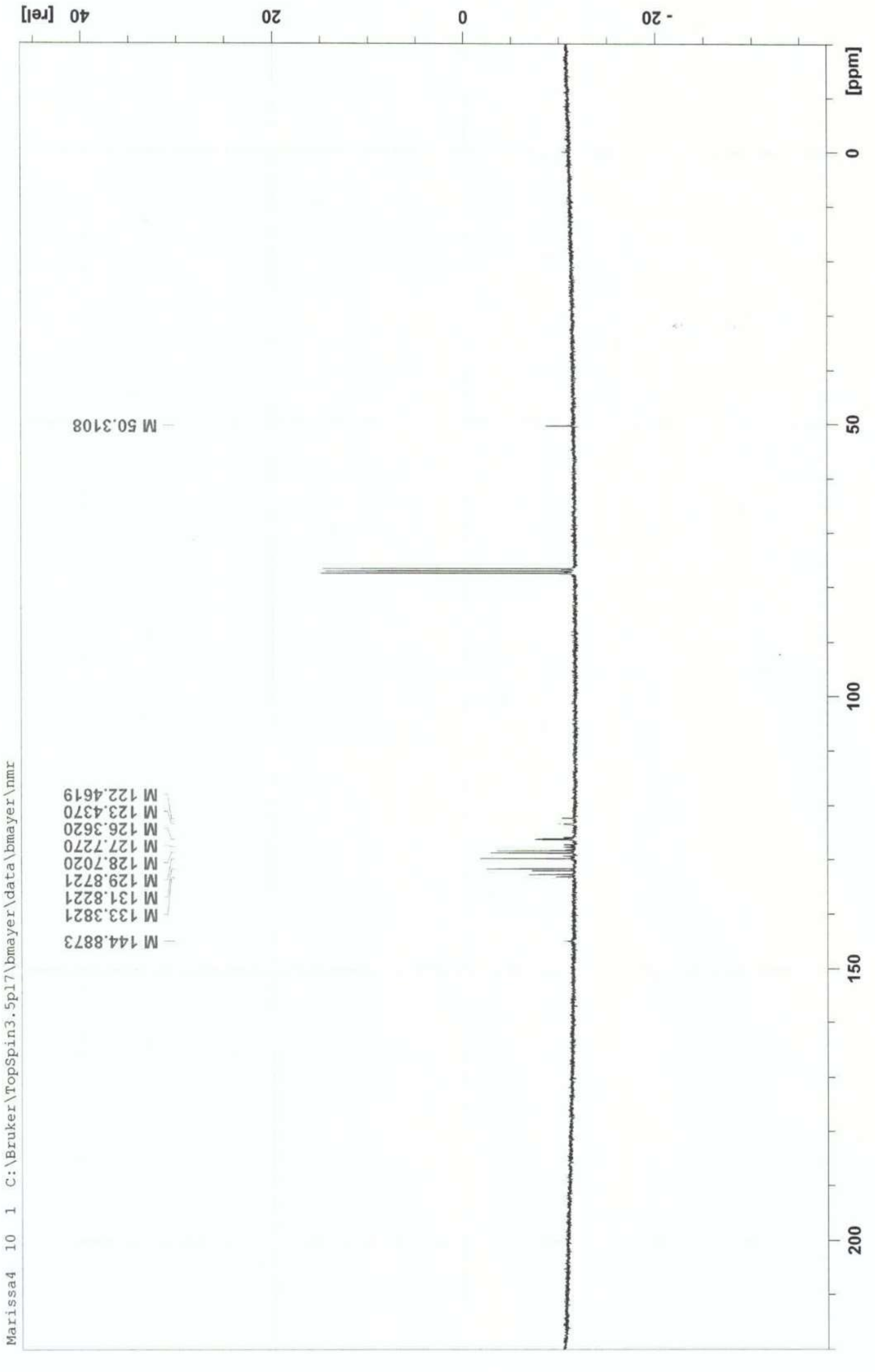

^{13}C NMR spectrum of **4**

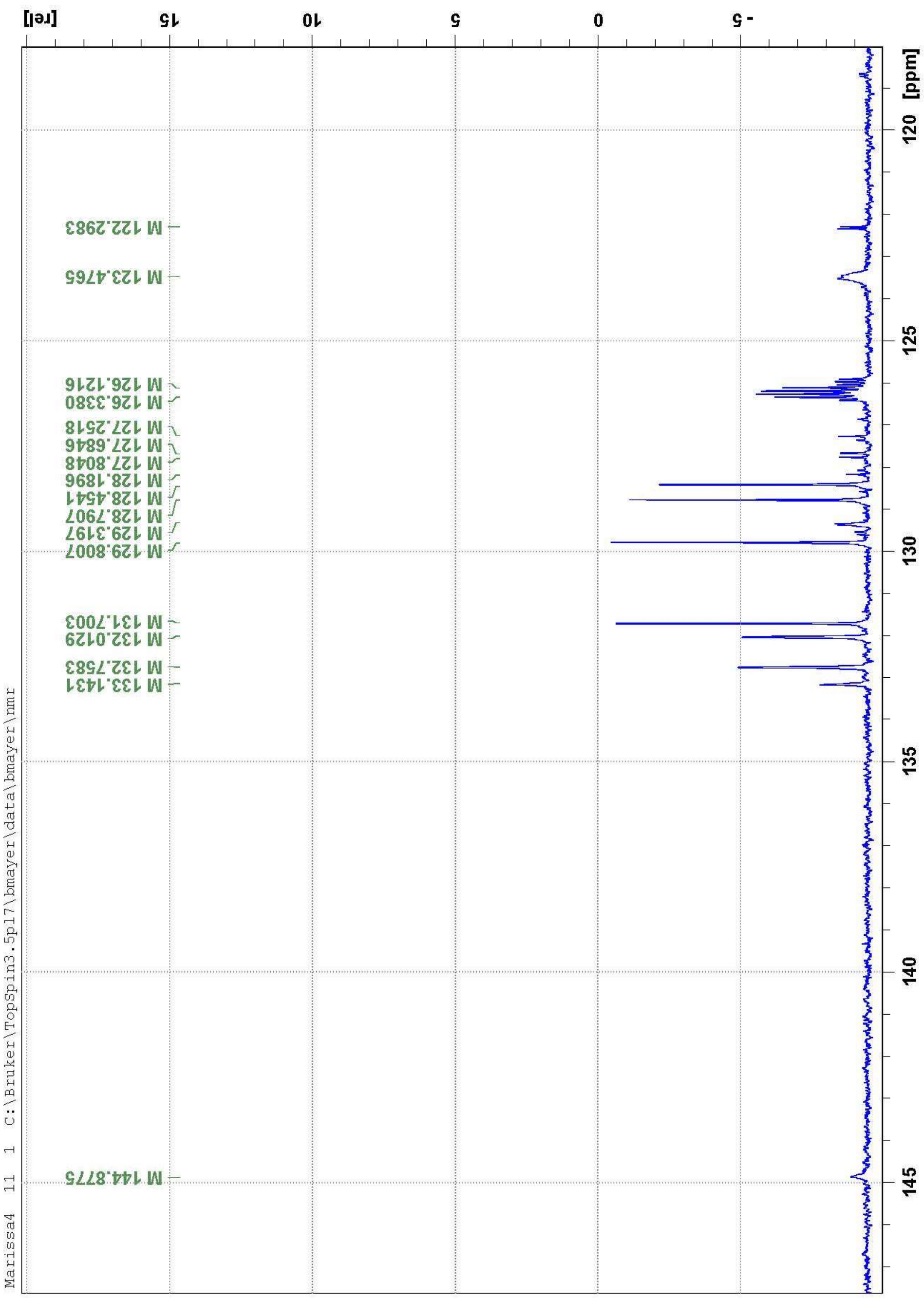

^{13}C NMR spectrum (expanded) of **4**

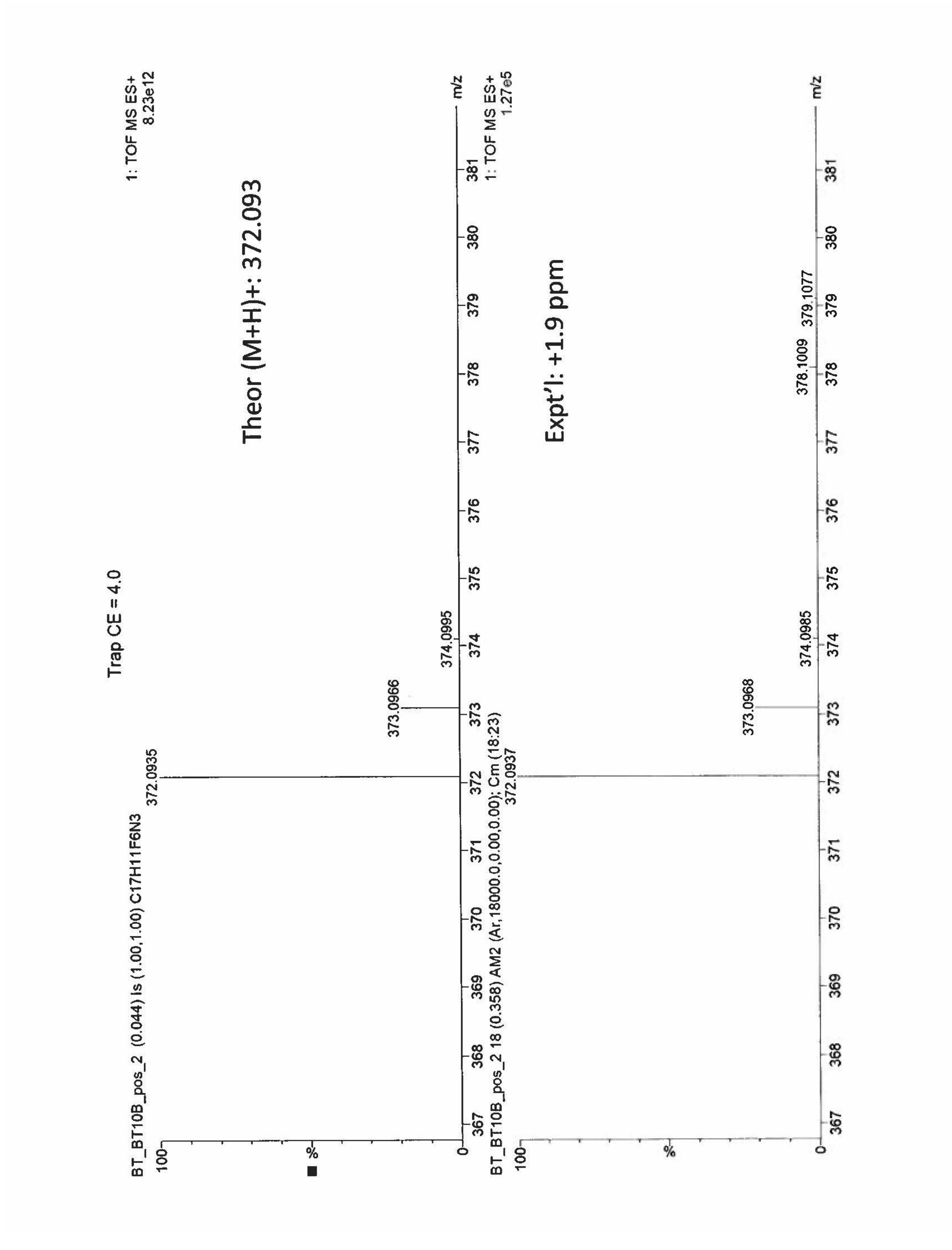

M + H+ HRMS data of **4**

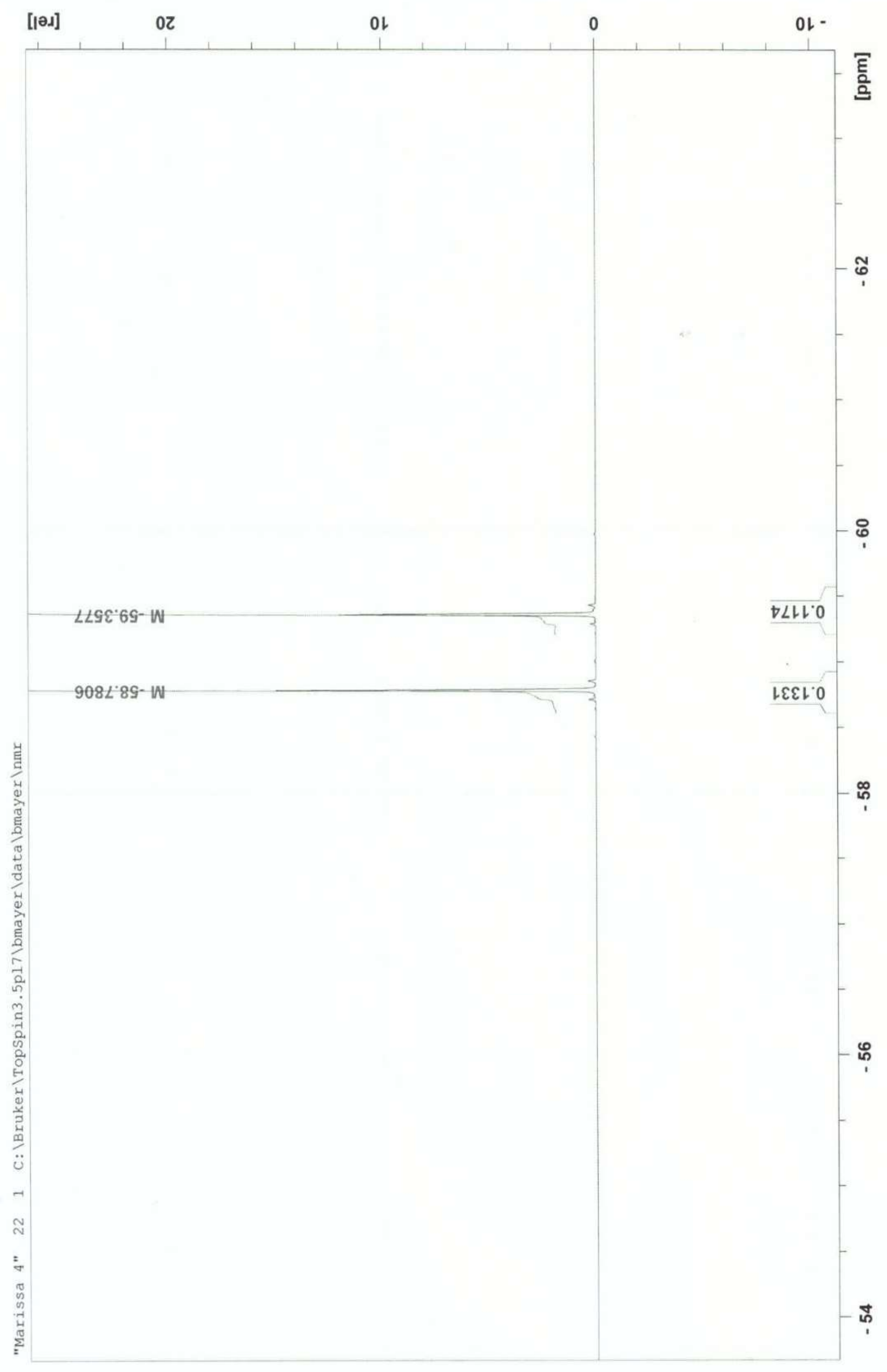

^{19}F NMR spectrum of **4**

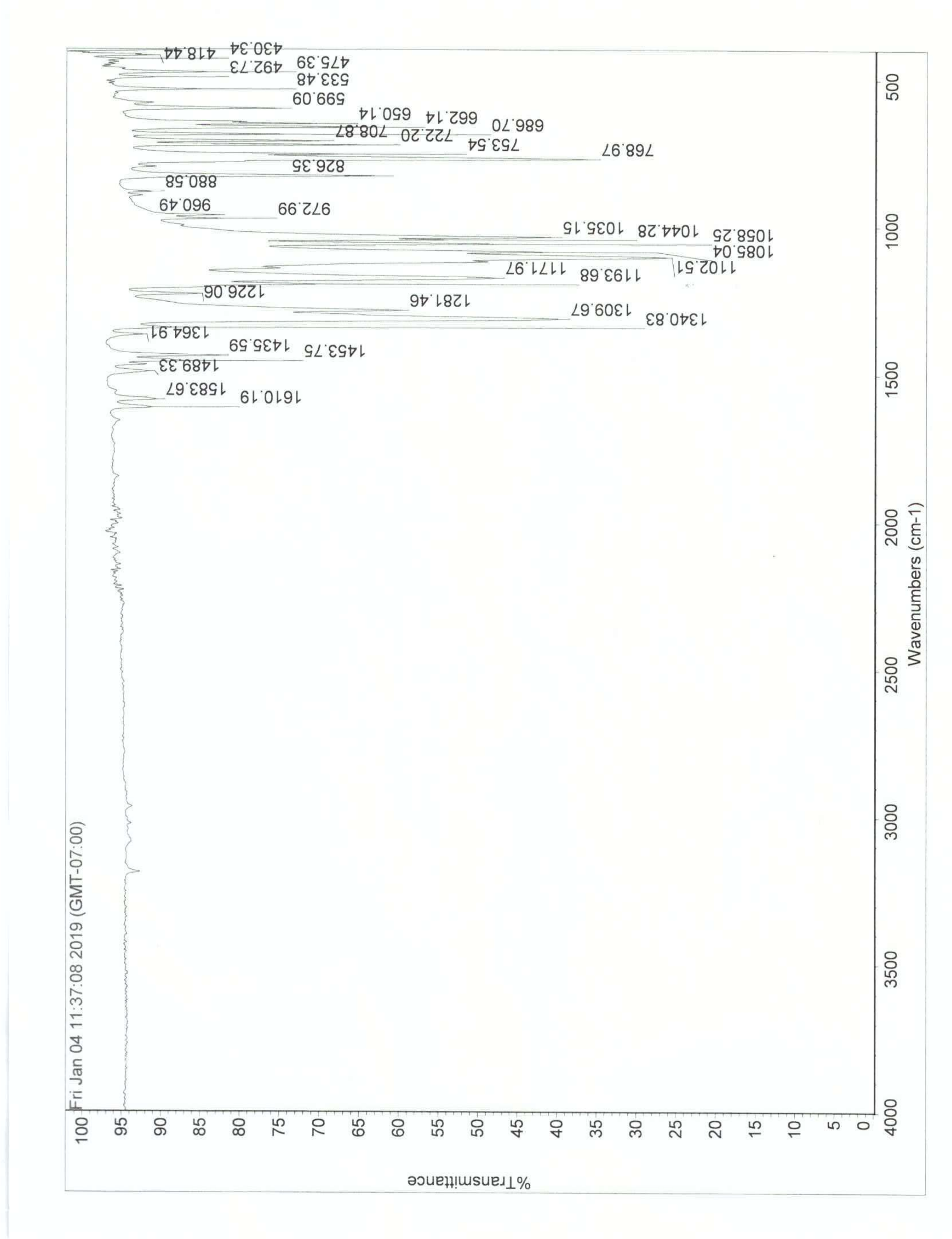

FTIR spectrum of **4**

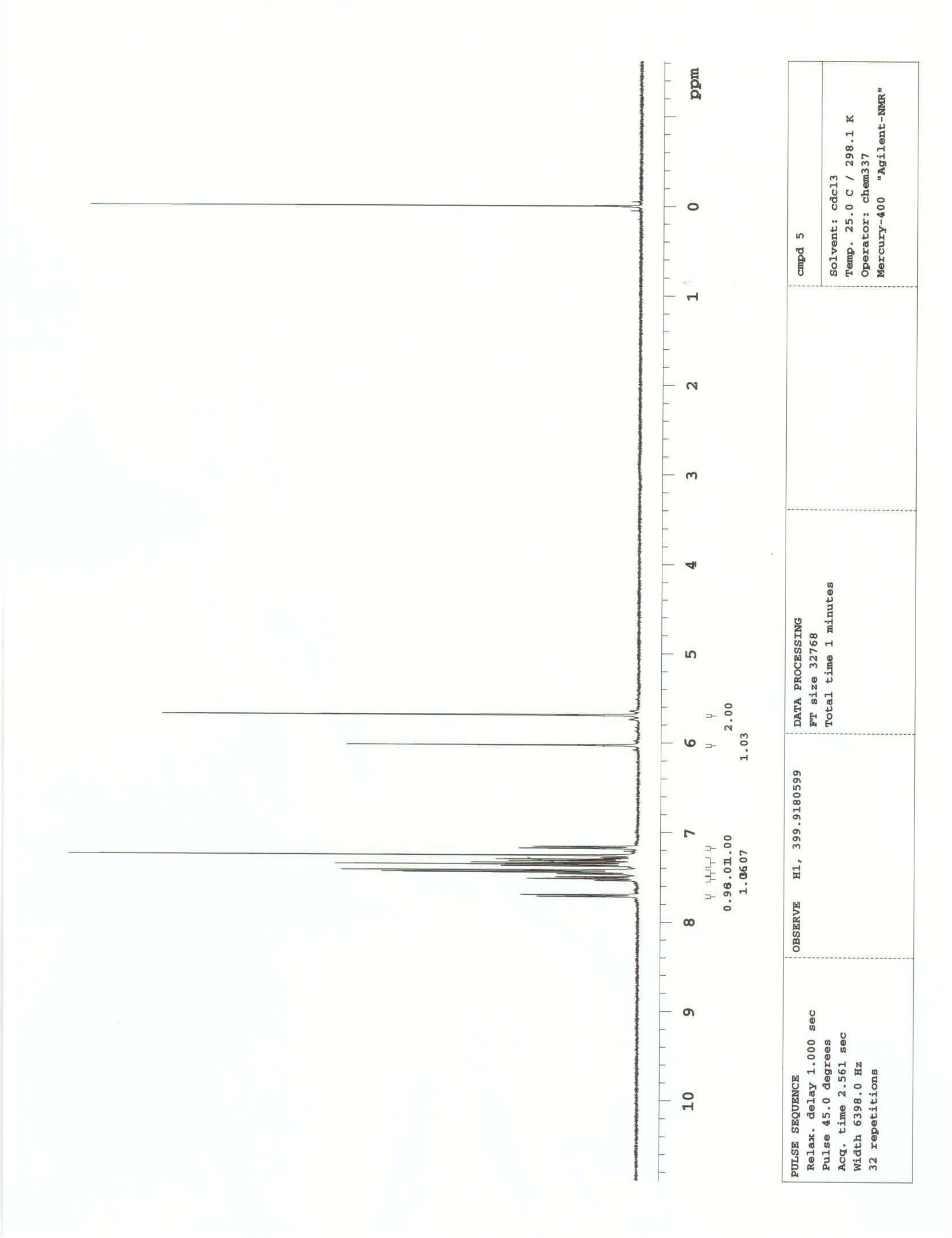

^{1}H NMR spectrum of **5**

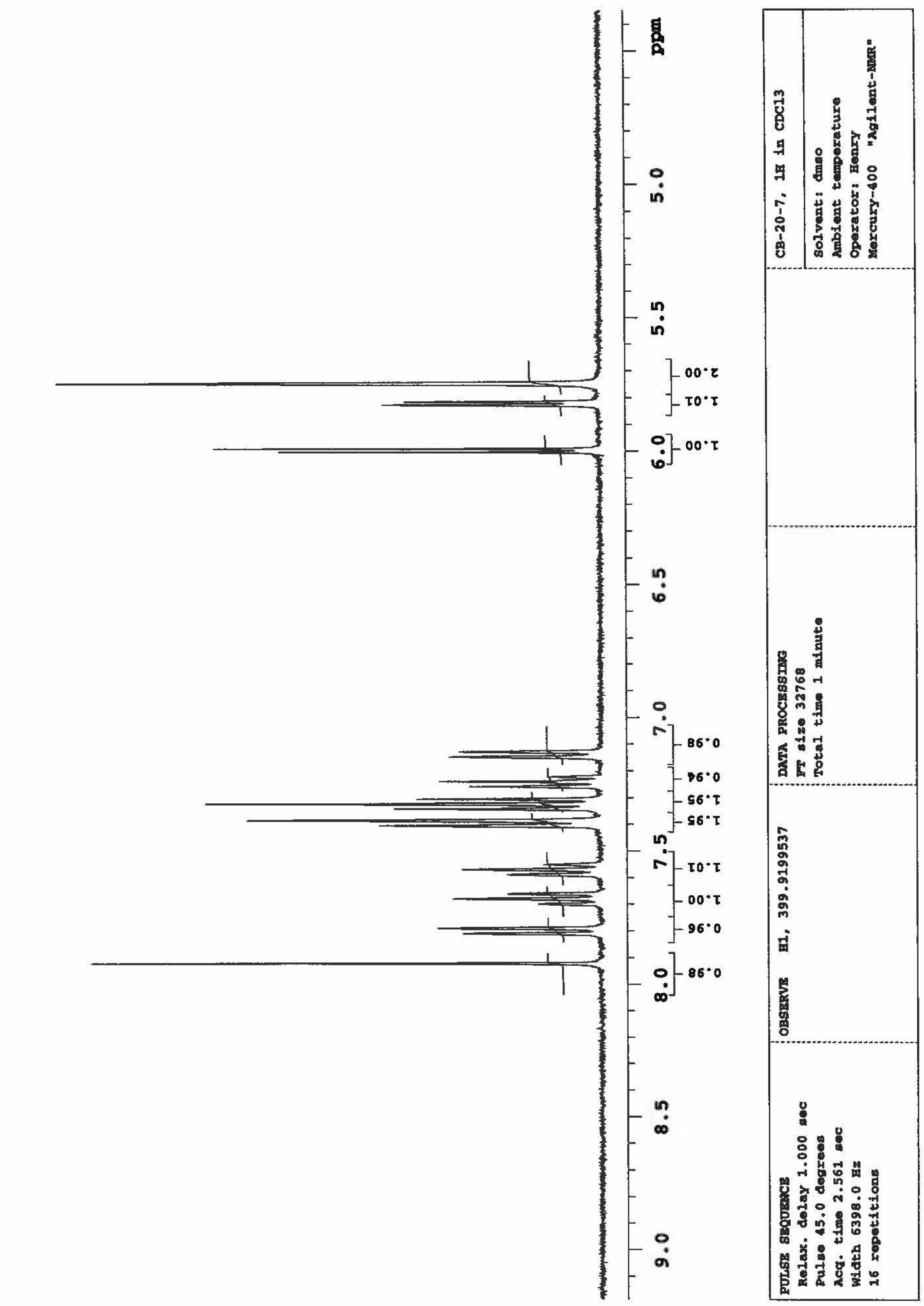

^{1}H NMR spectrum (expanded) of **5**

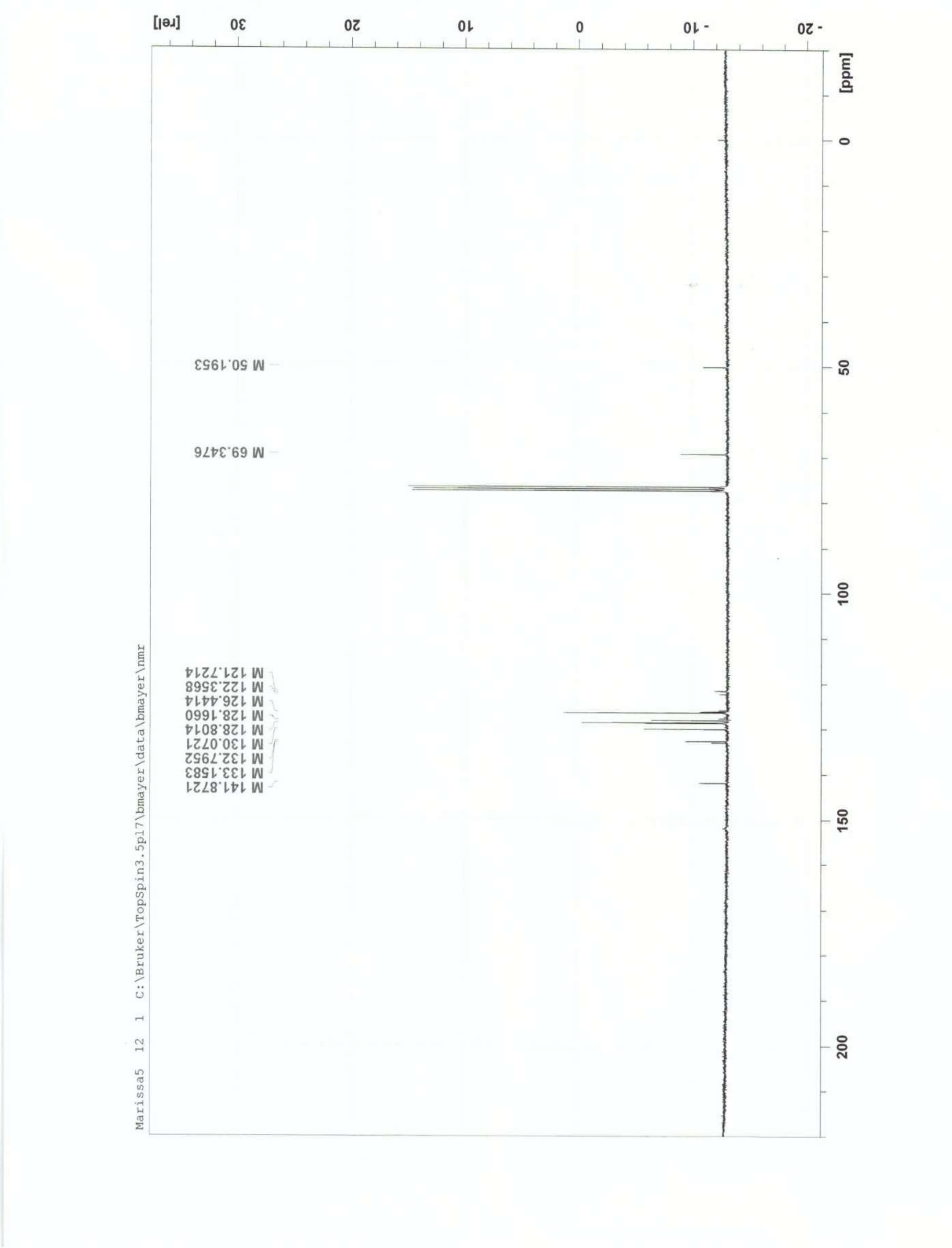

^{13}C NMR spectrum of **5**

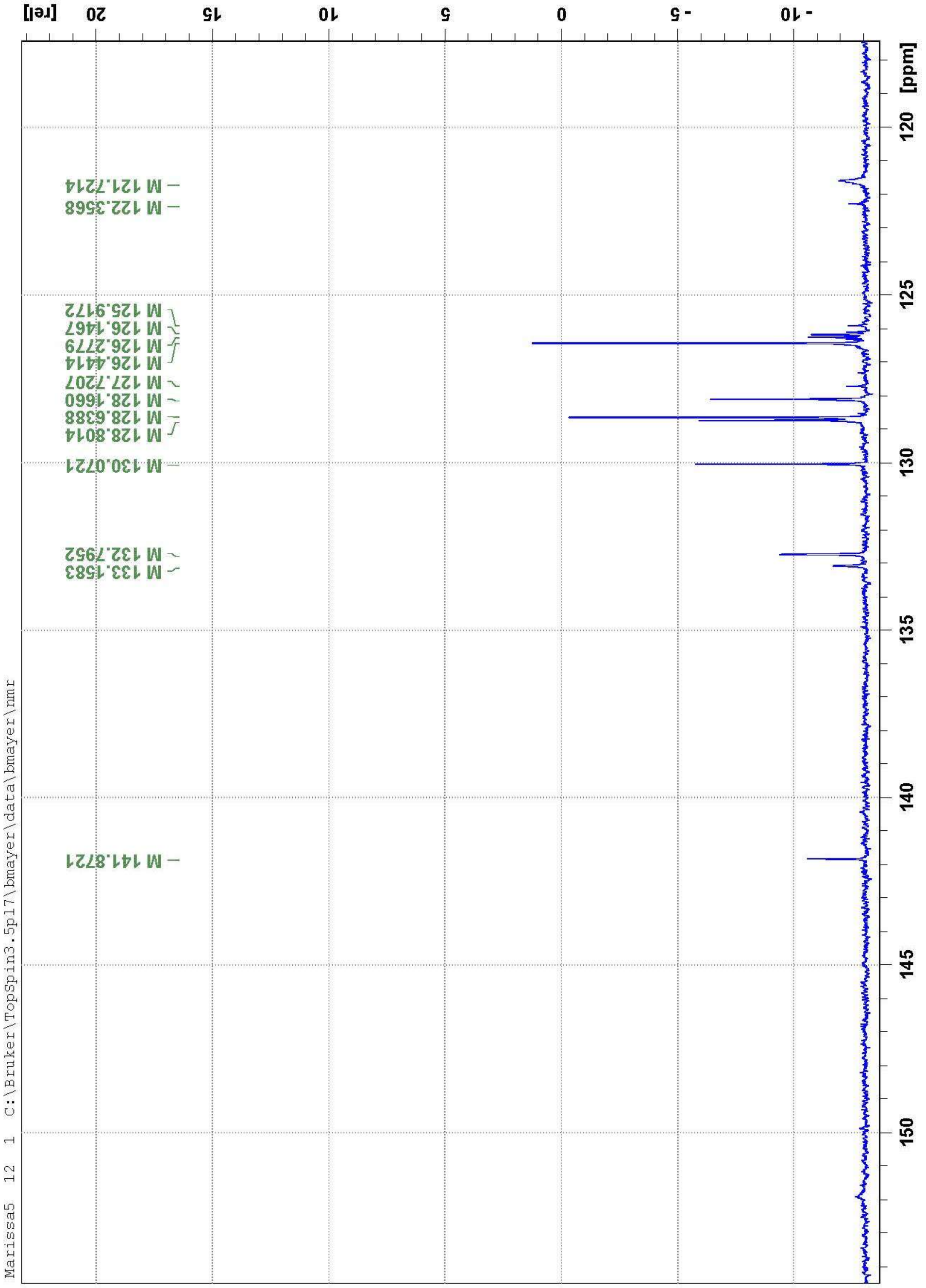

^{13}C NMR spectrum (expanded) of **5**

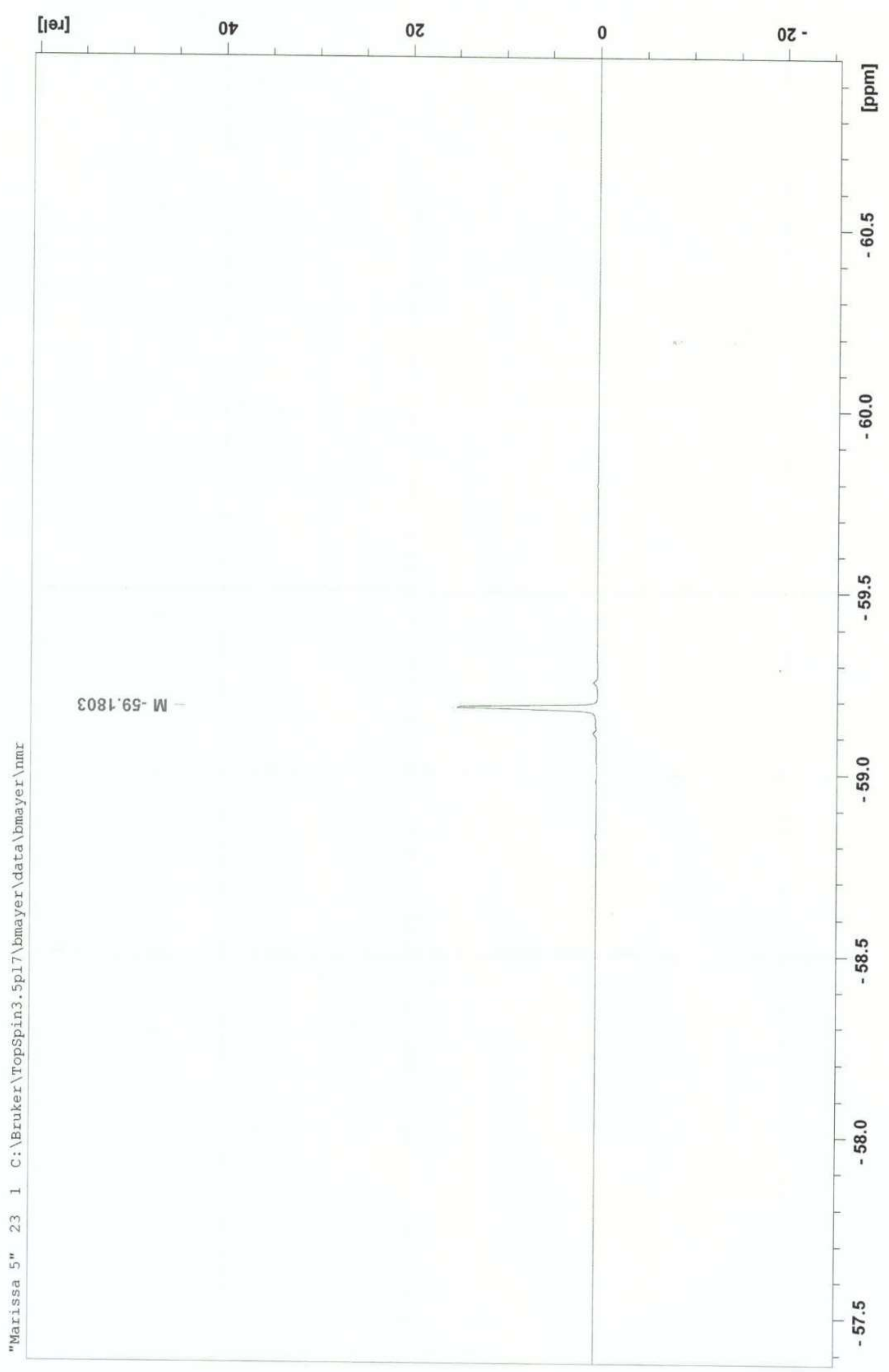

^{19}F NMR spectrum of **5**

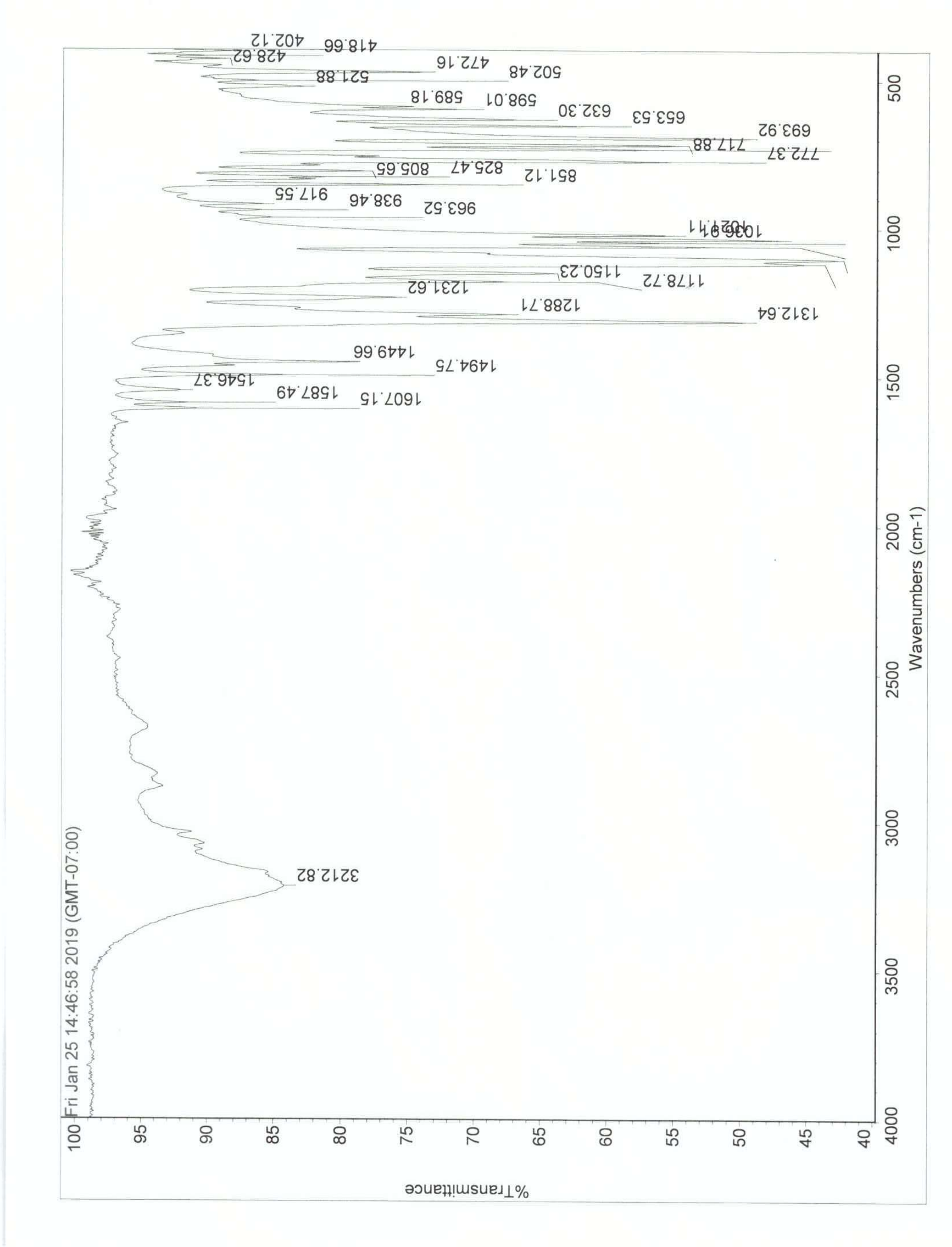

FTIR spectrum of **5**

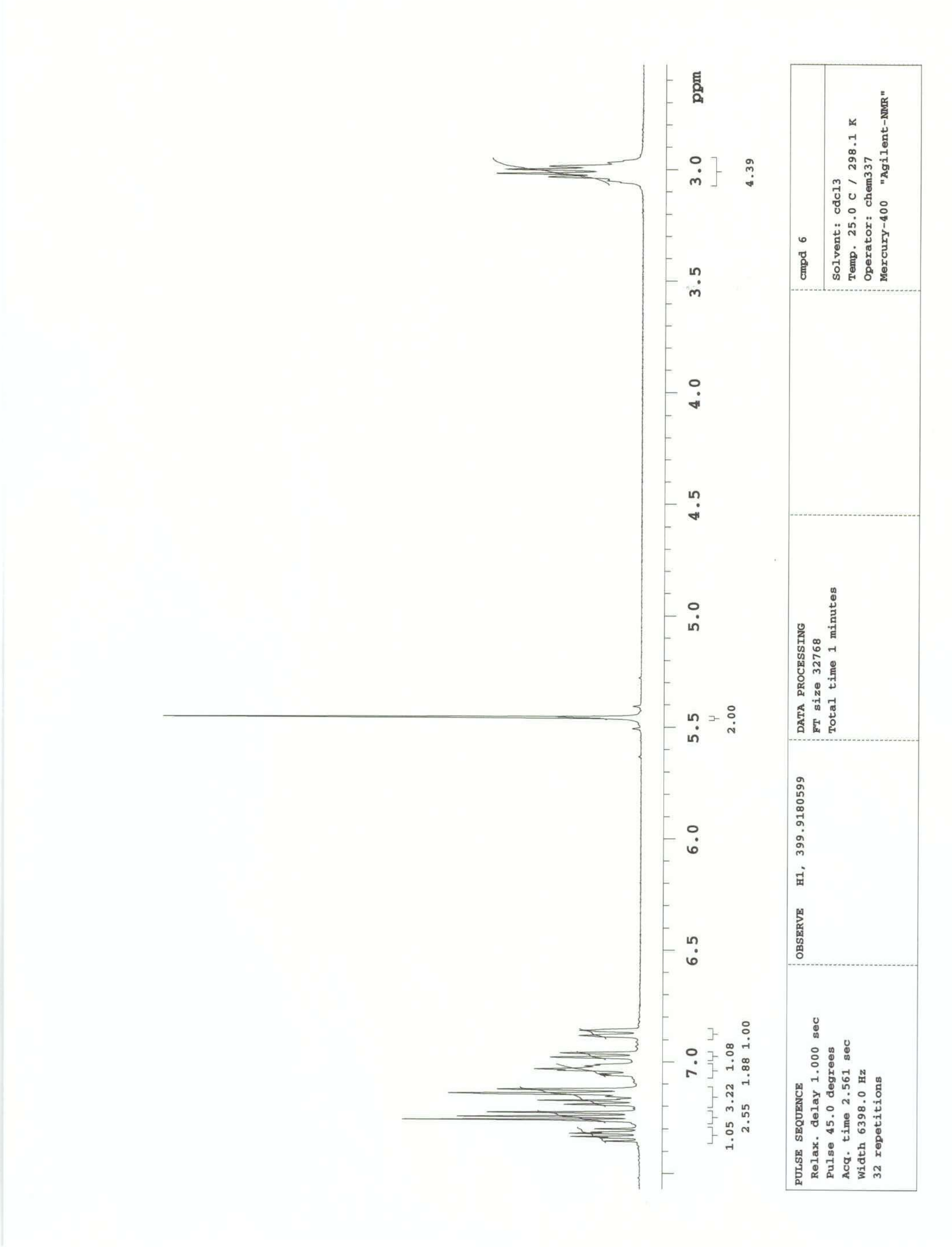

^{1}H NMR spectrum of **6**

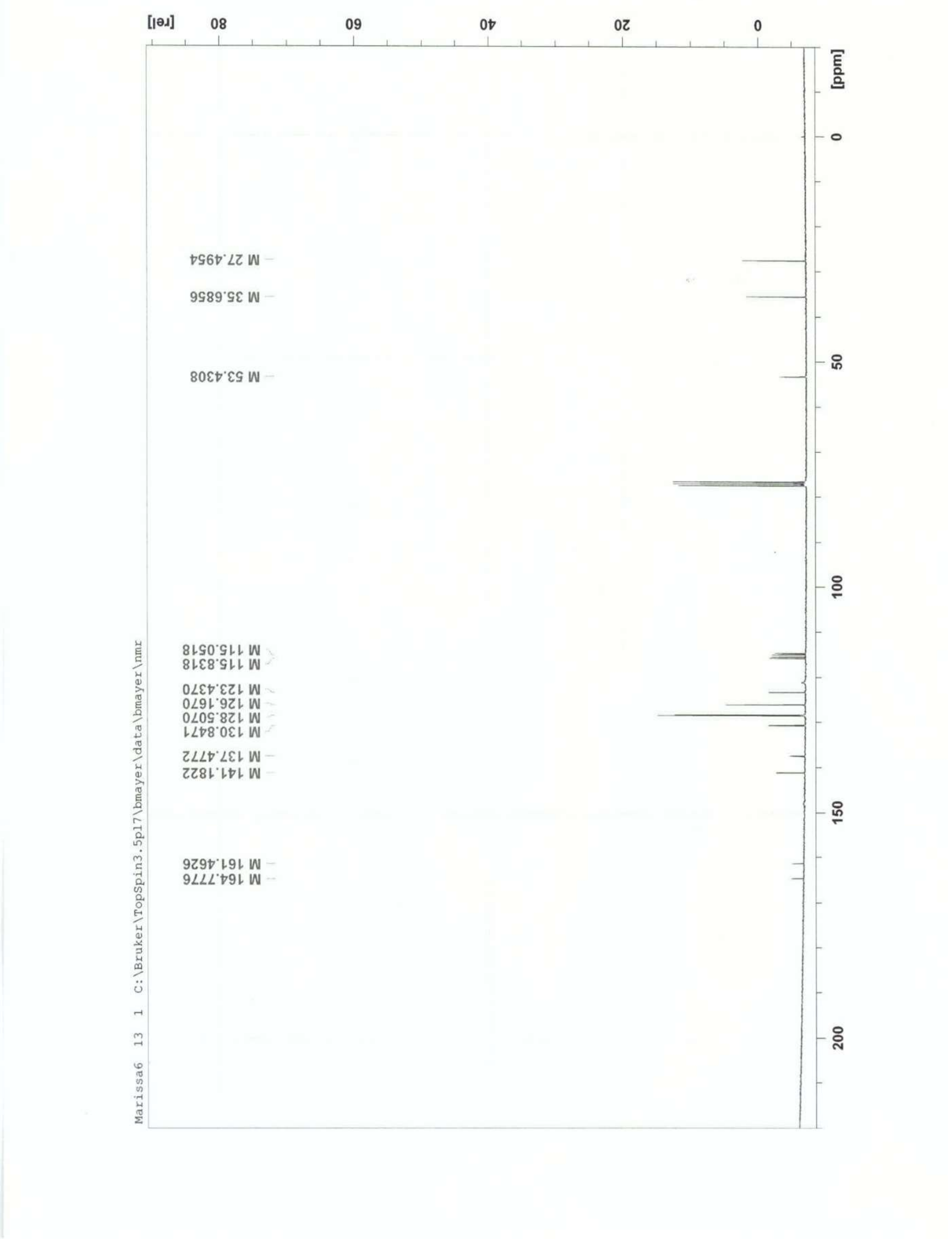

^{13}C NMR spectrum of **6**

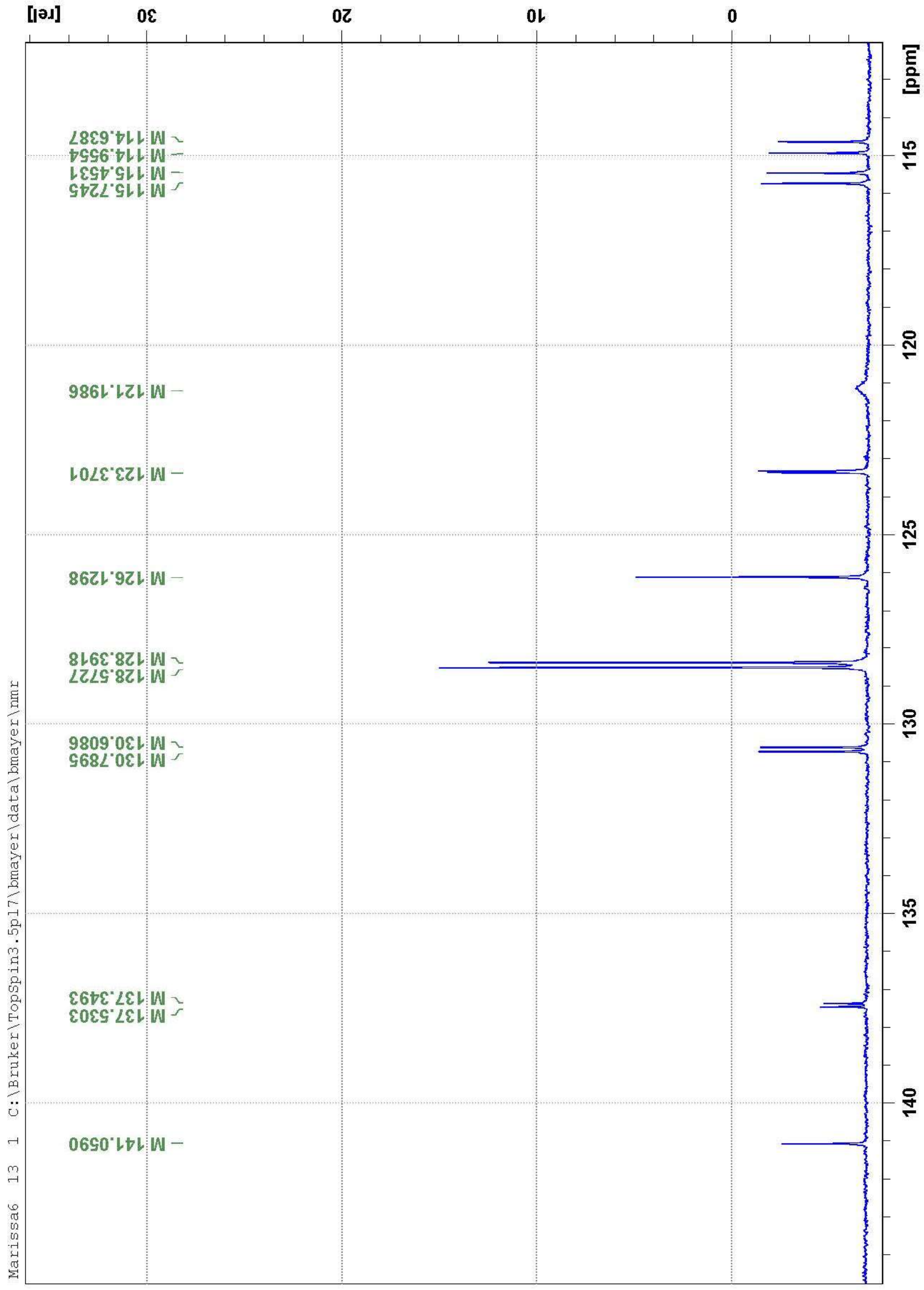

^{13}c NMR spectrum (expanded) of **6**

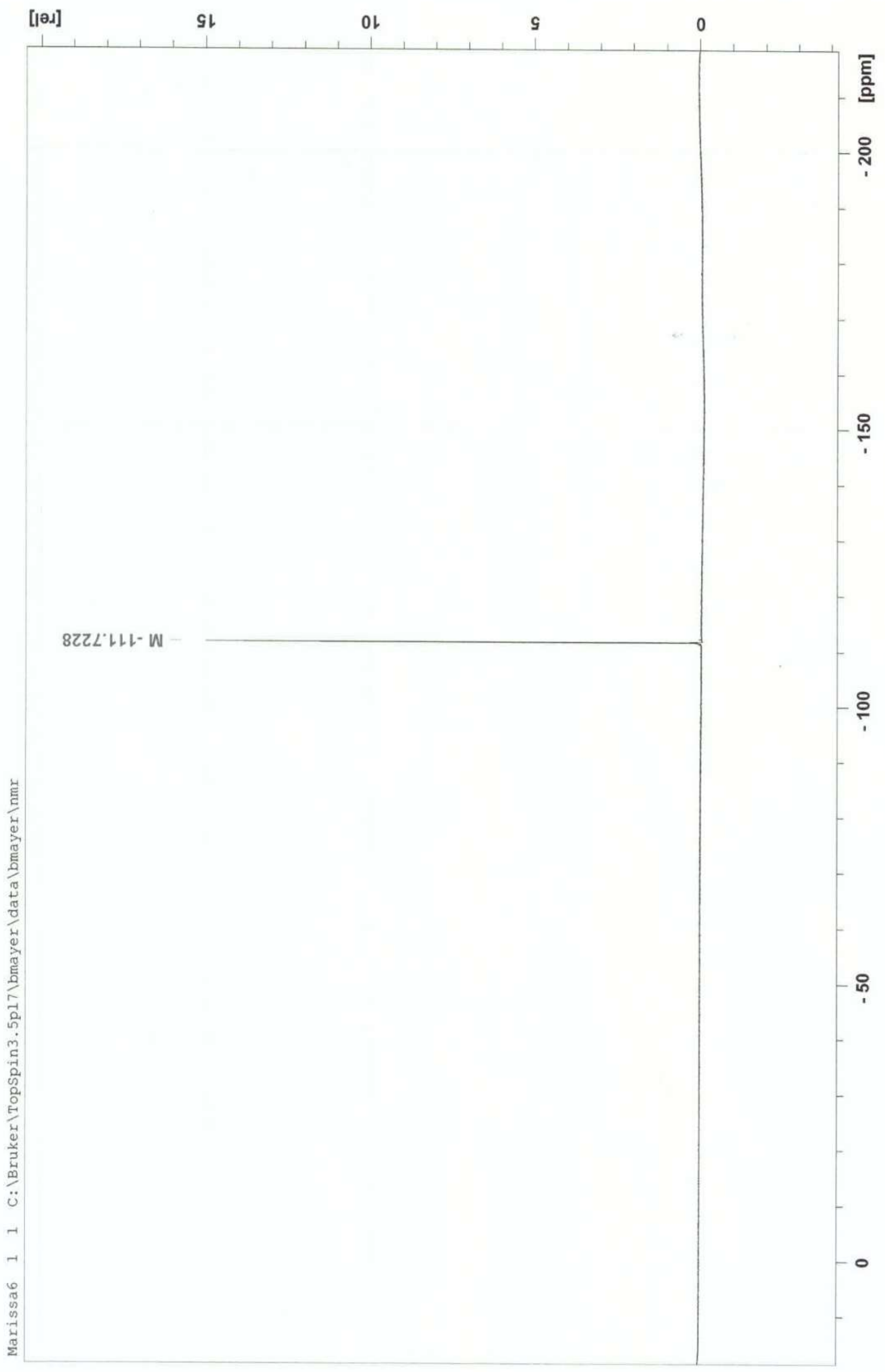

^{19}F NMR spectrum of **6**

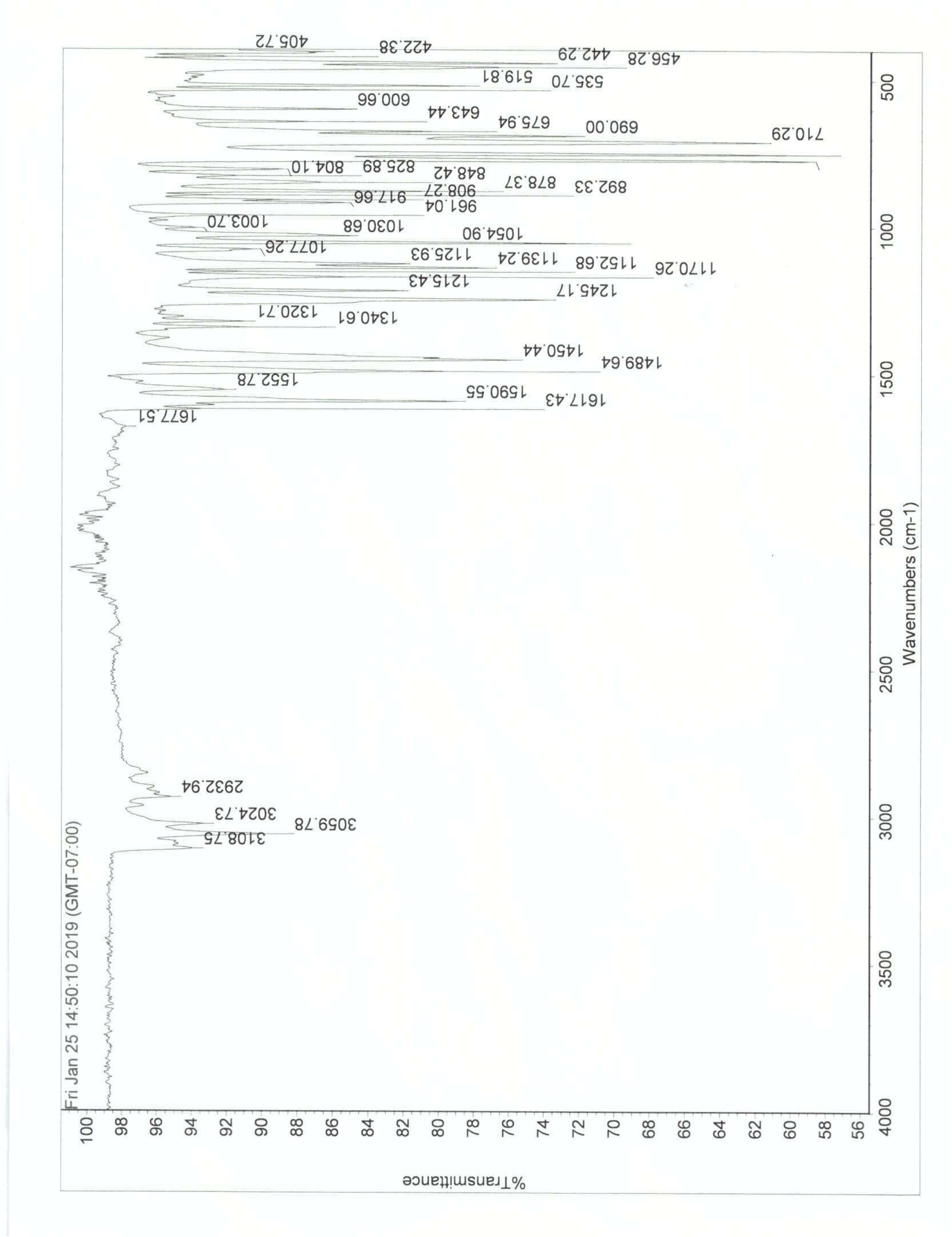

FTIR spectrum of **6**

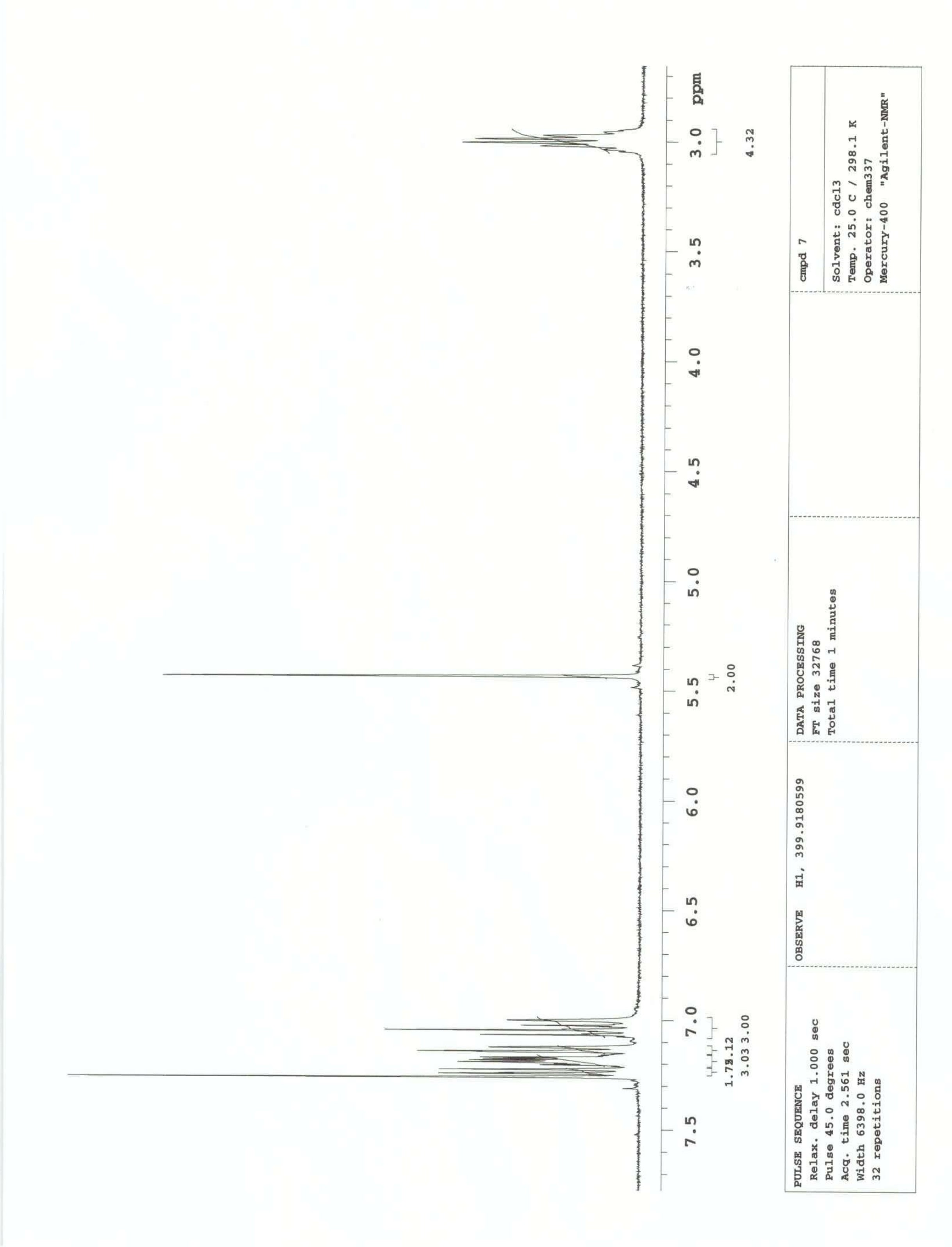

^{1}H NMR spectrum of **7**

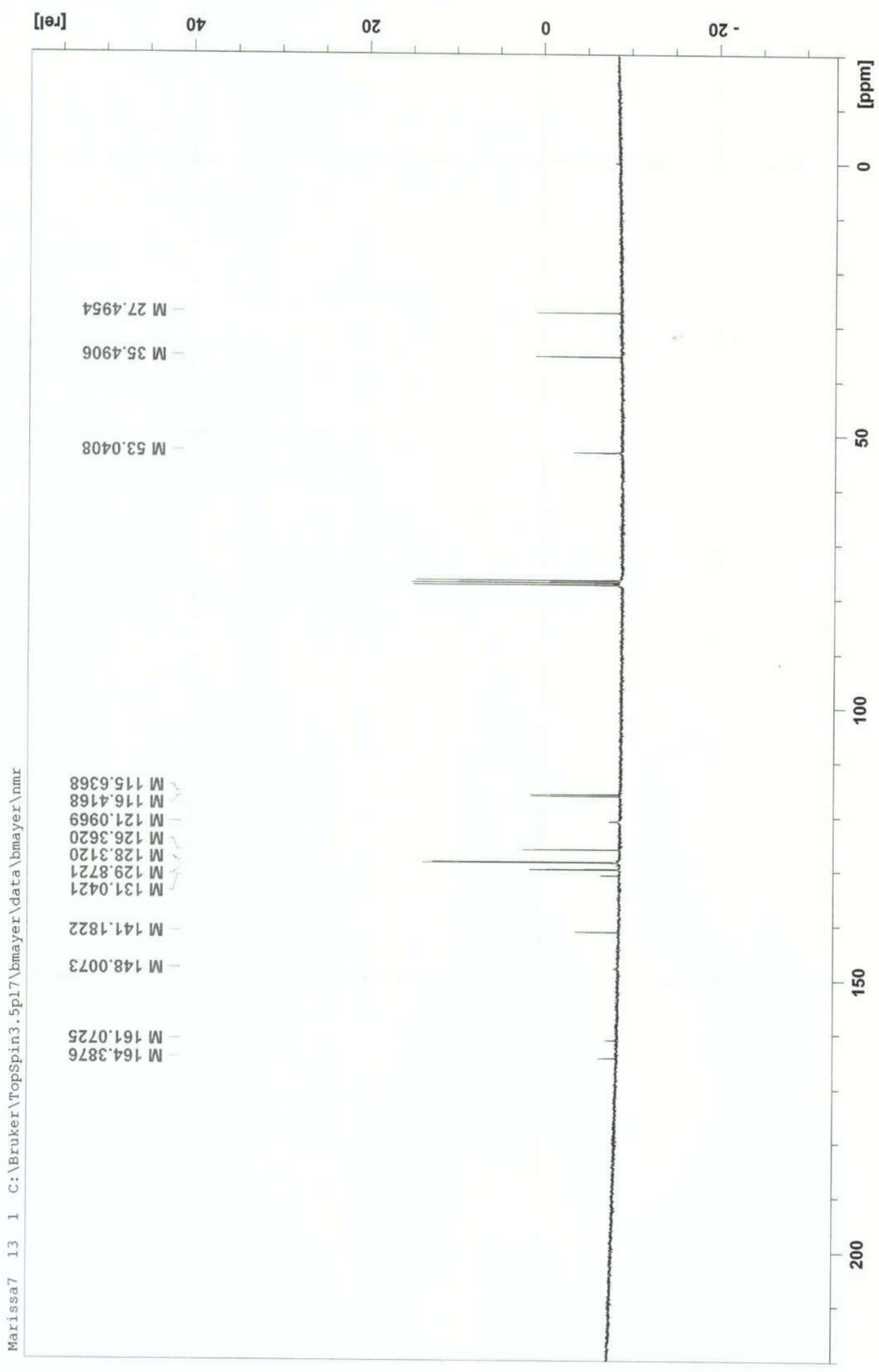

^{13}C NMR spectrum of **7**

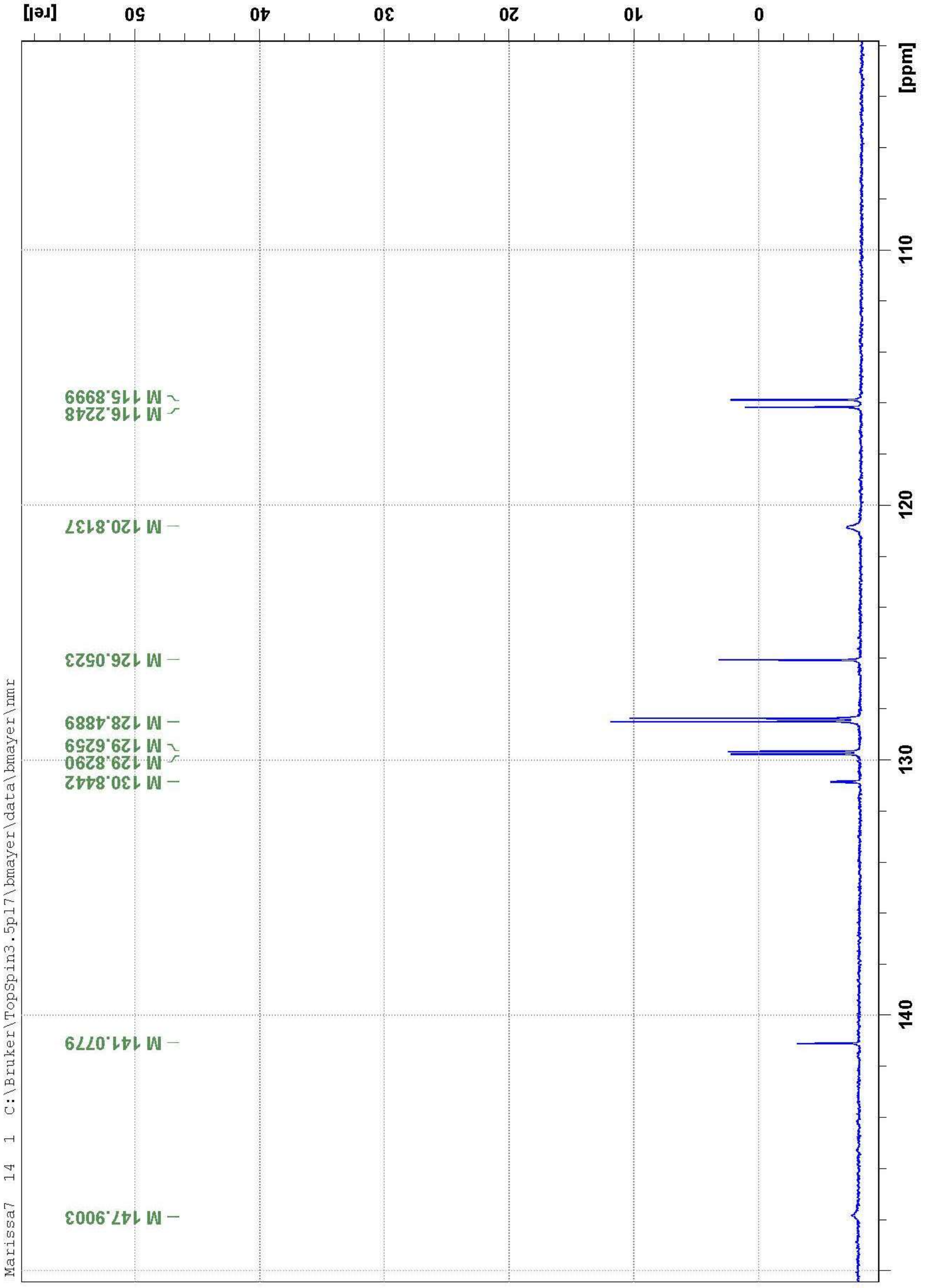

^{13}C NMR spectrum (expanded) of **7**

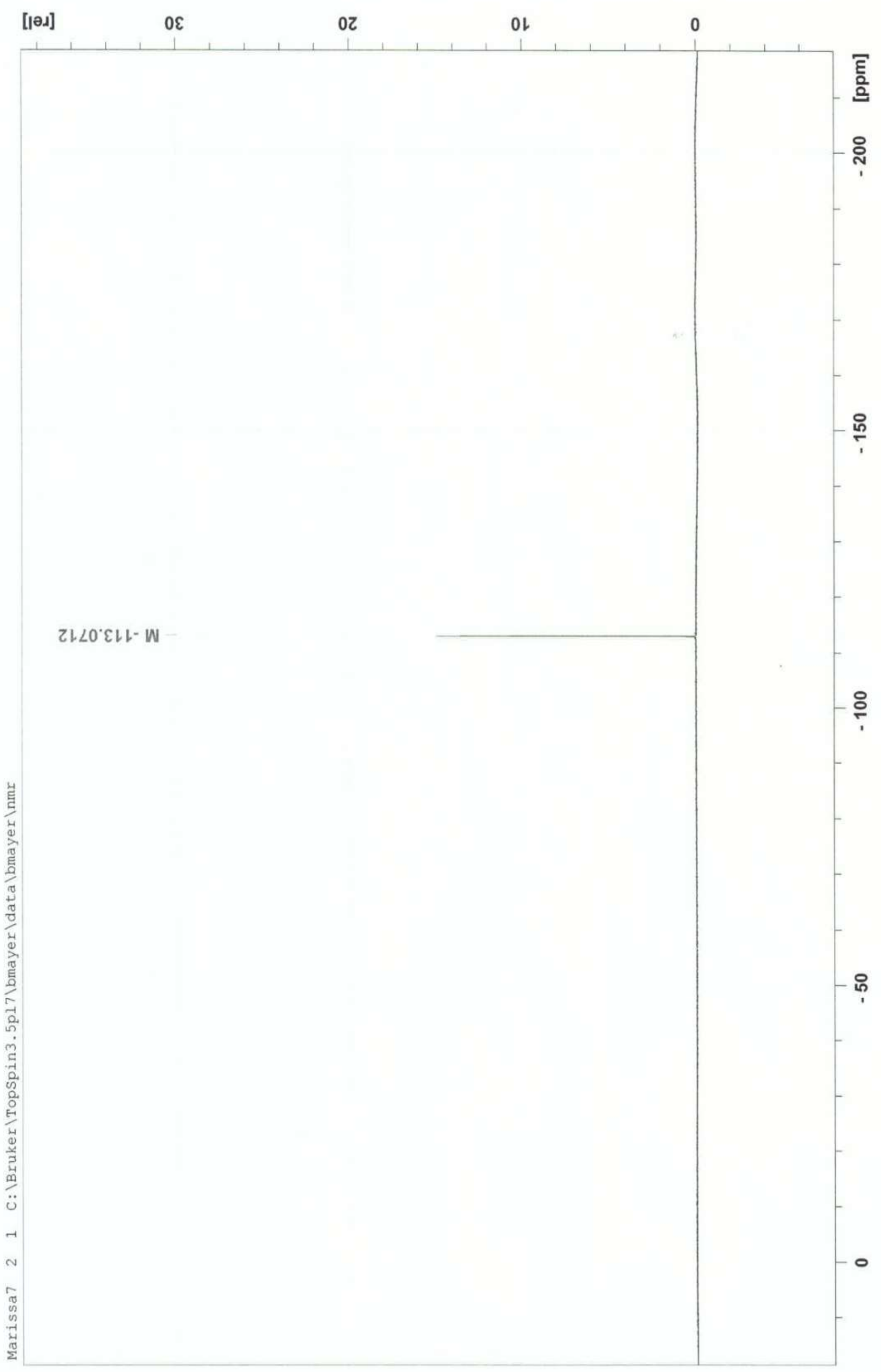

^{19}F NMR spectrum of **7**

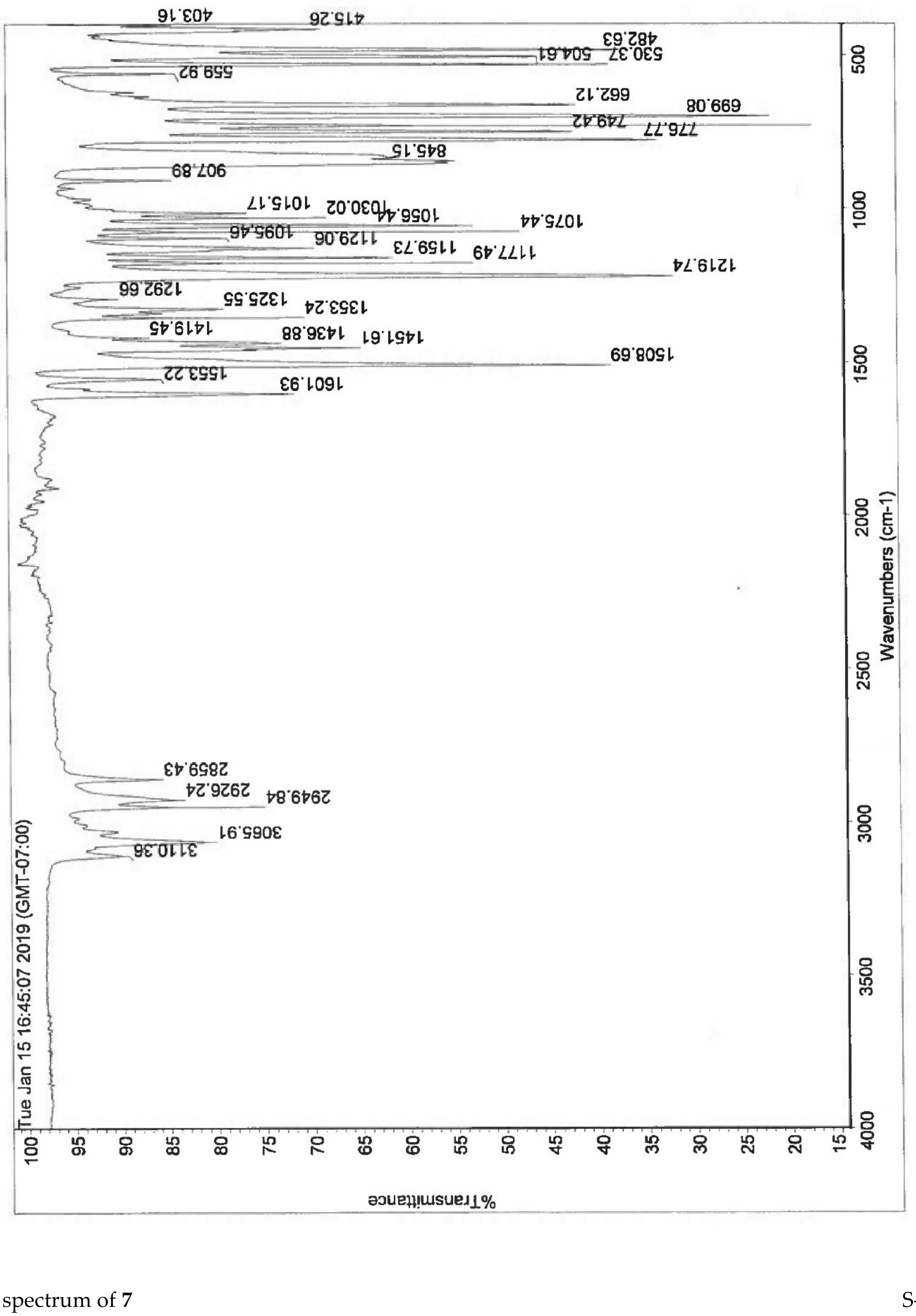

FTIR spectrum of **7**

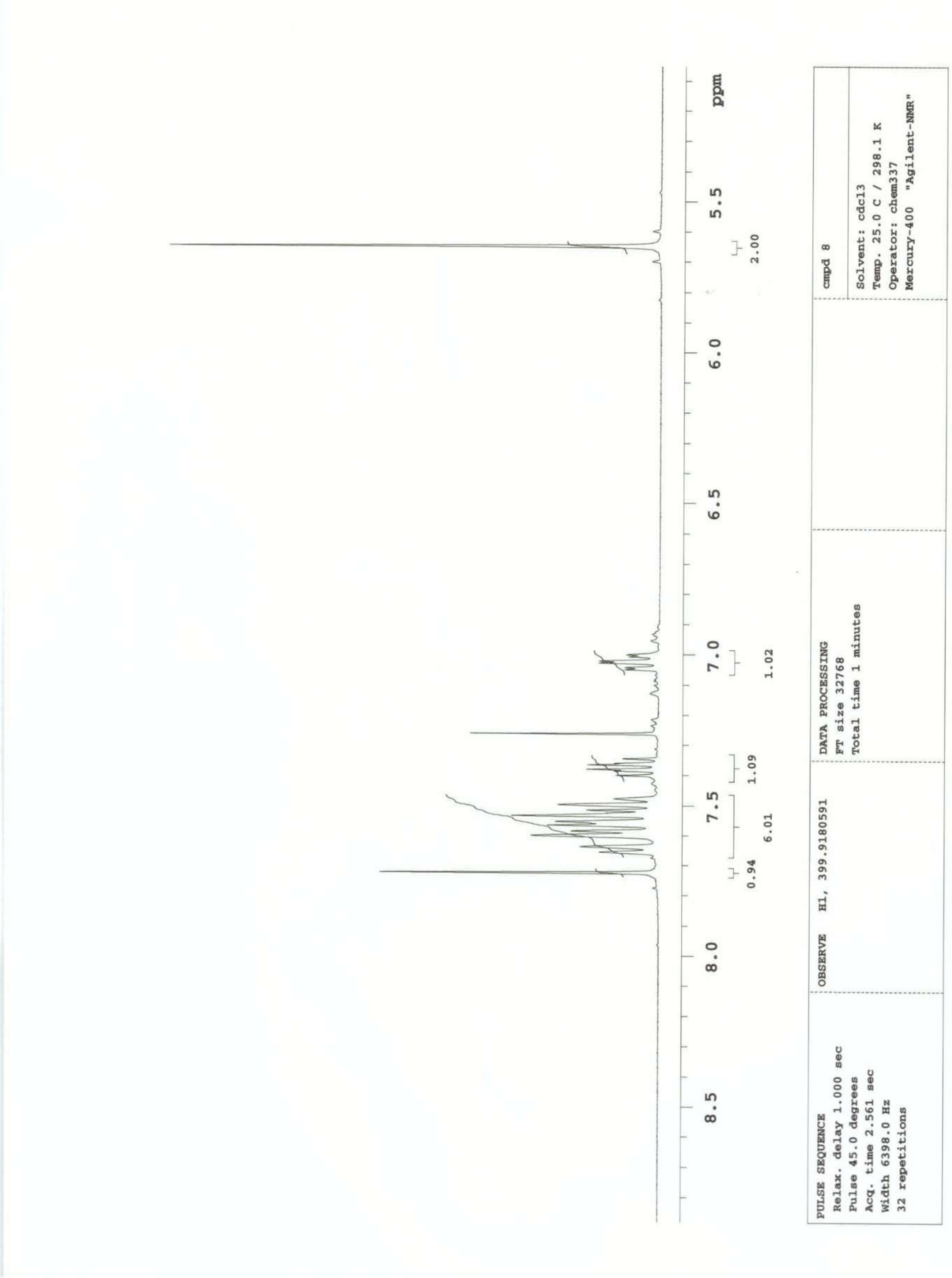

^{1}H NMR spectrum of **8**

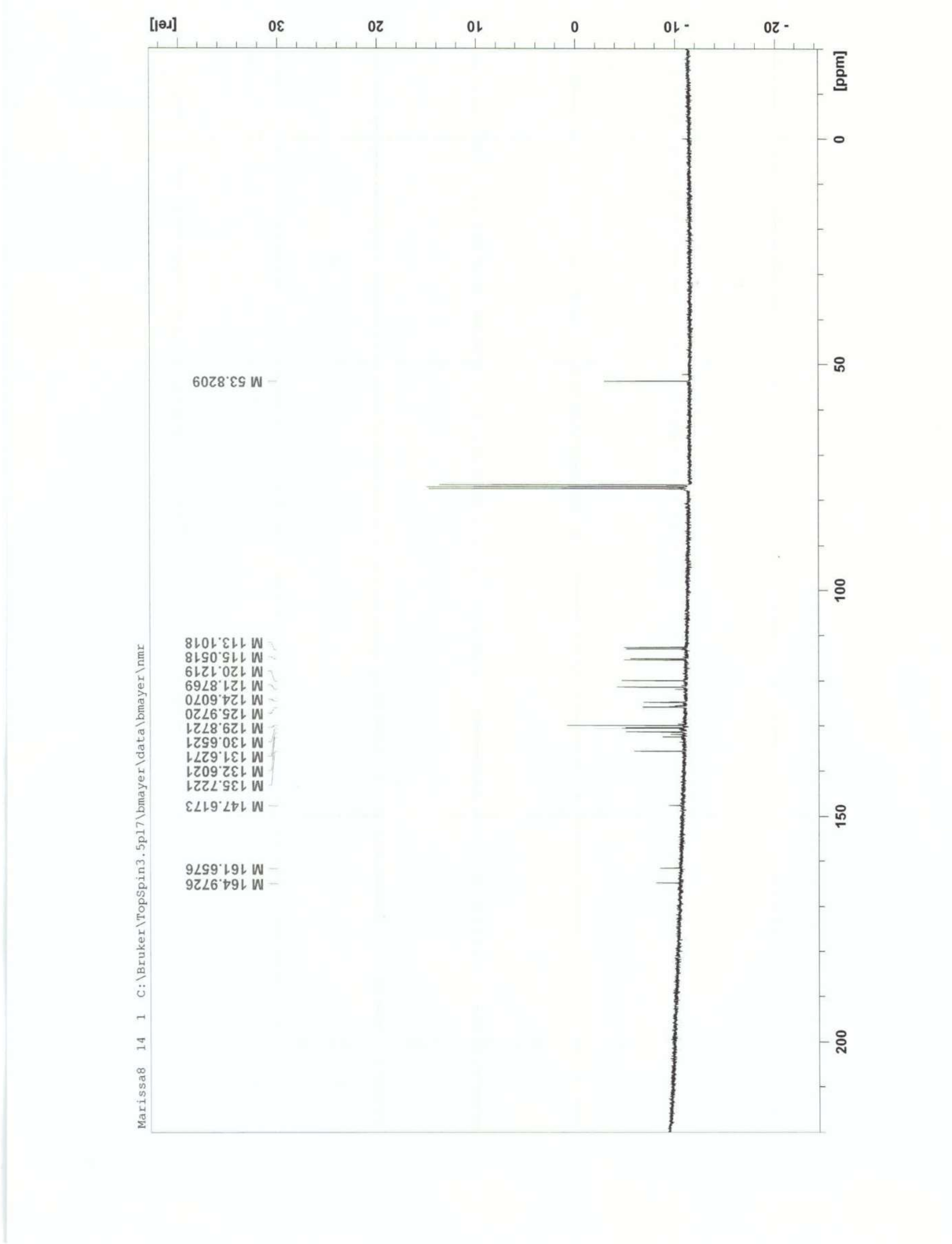

^{13}C NMR spectrum of **8**

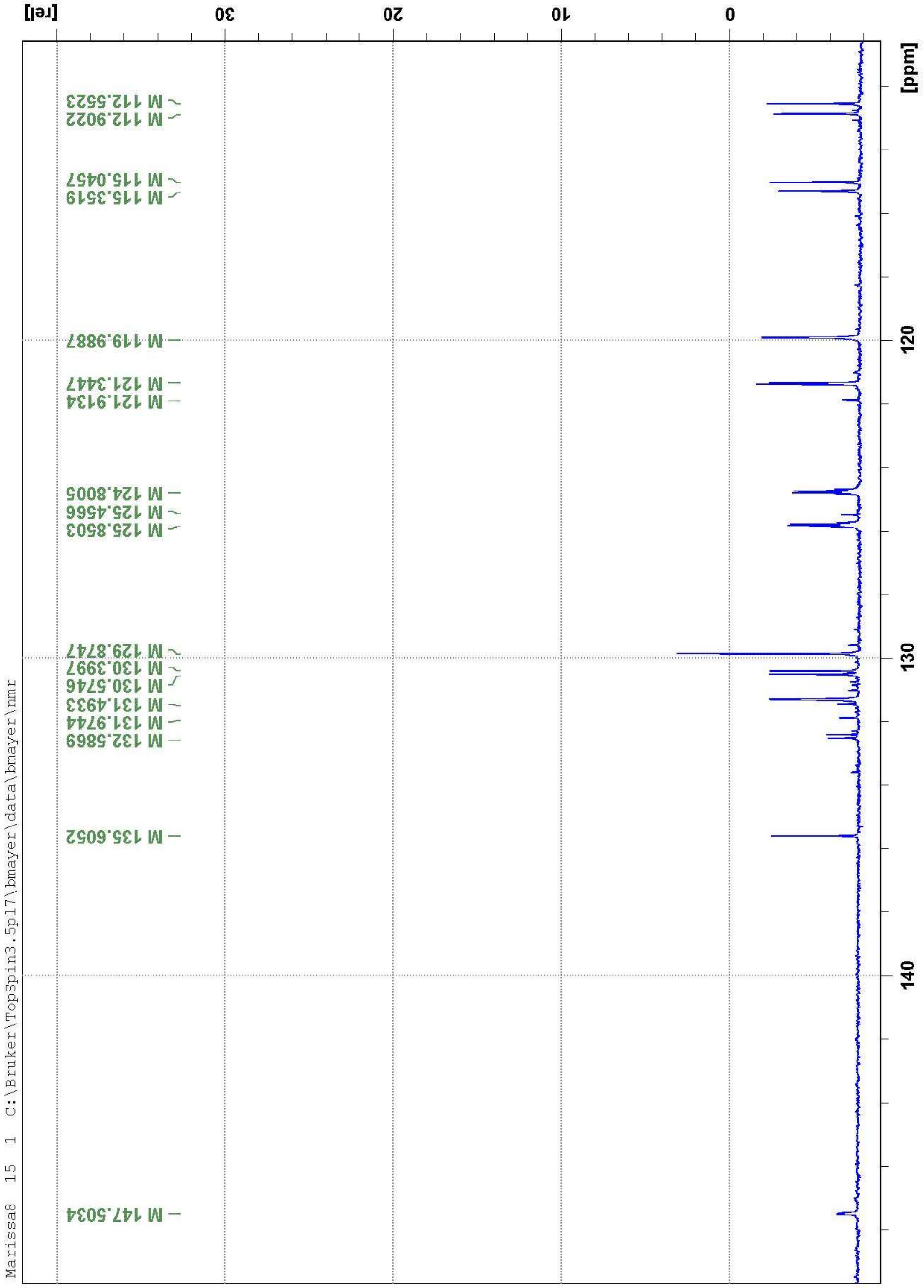

^{13}C NMR spectrum (expanded) of **8**

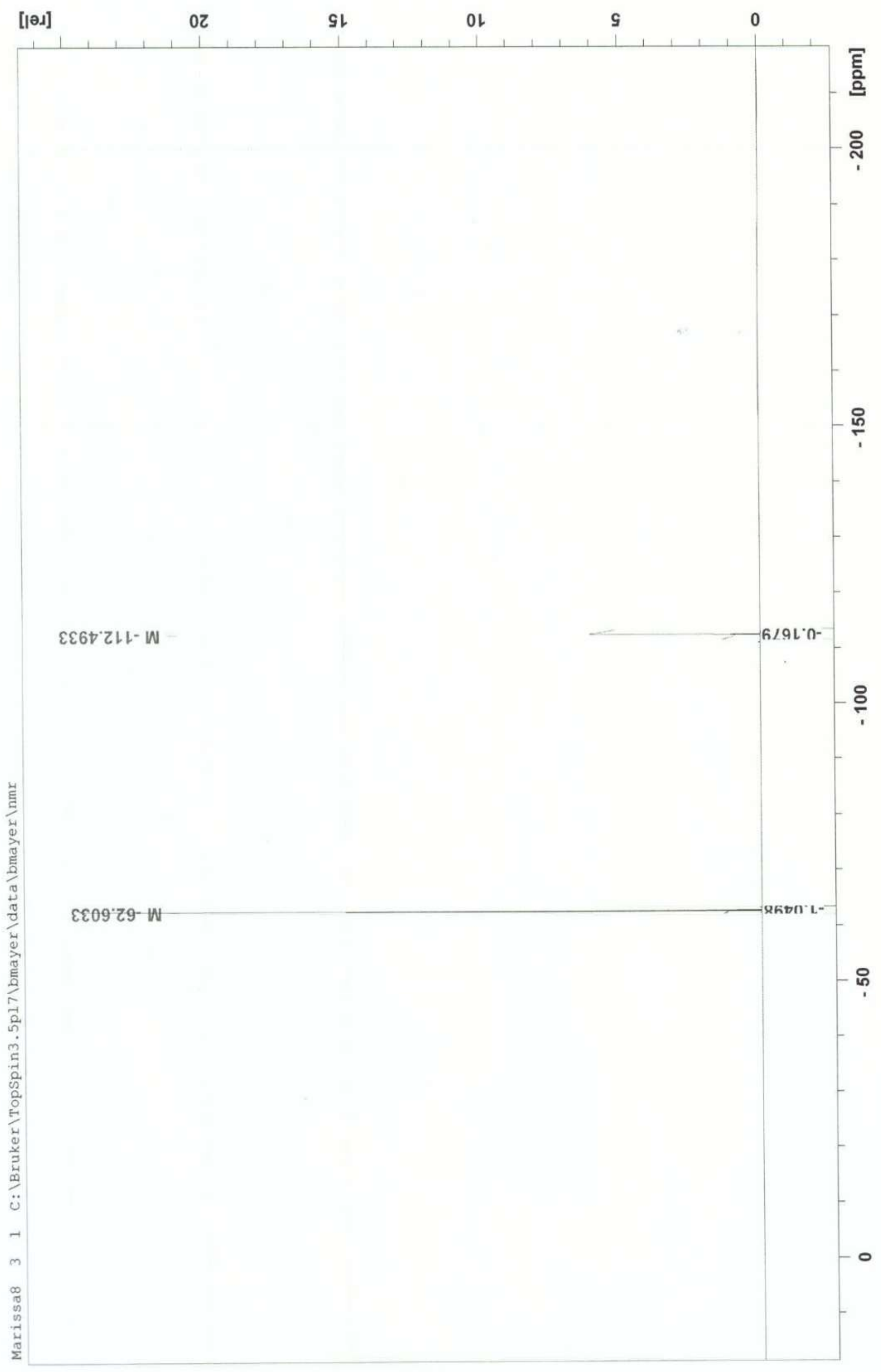

^{19}F NMR spectrum of **8**

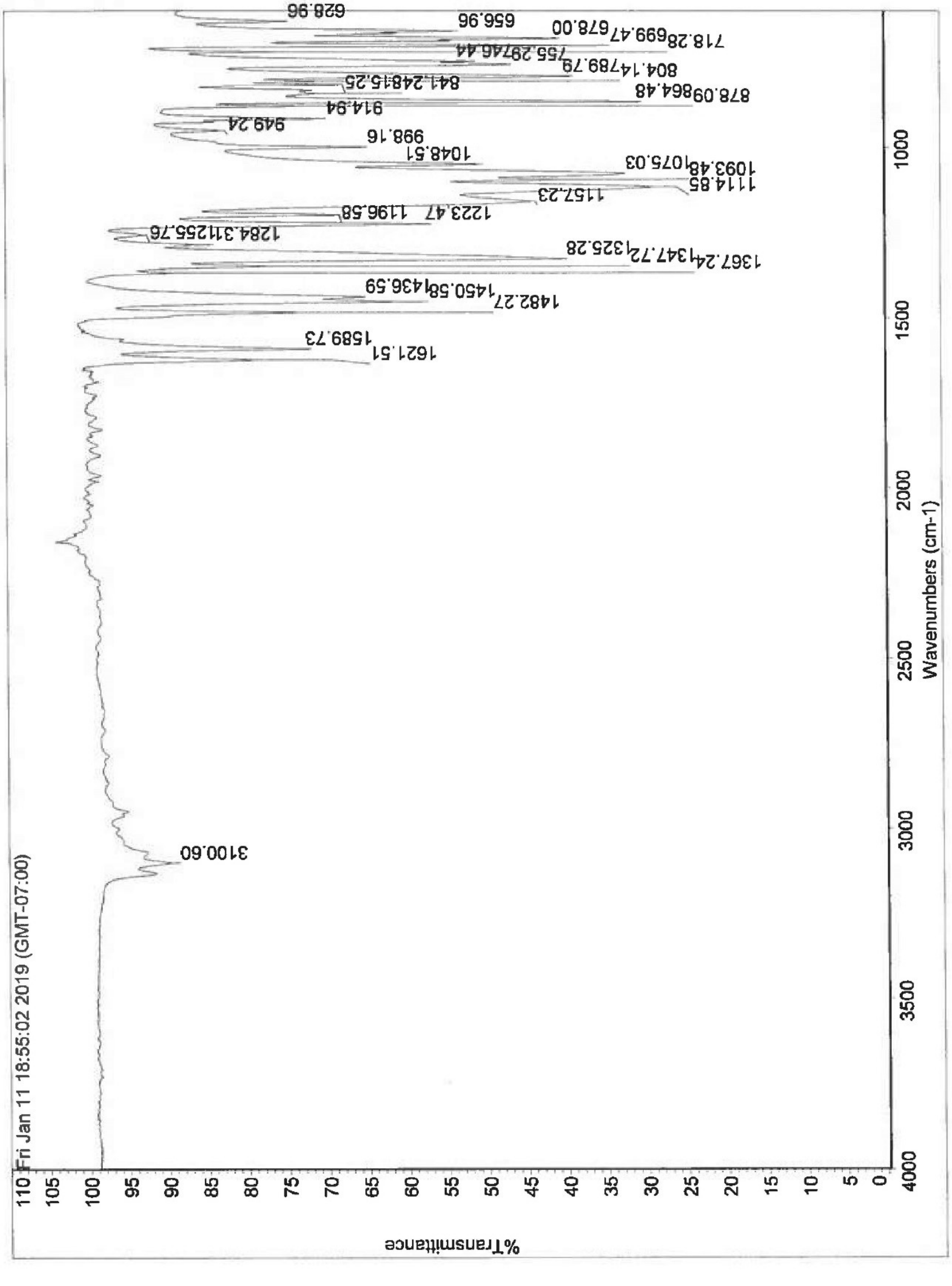

FTIR spectrum of **8**

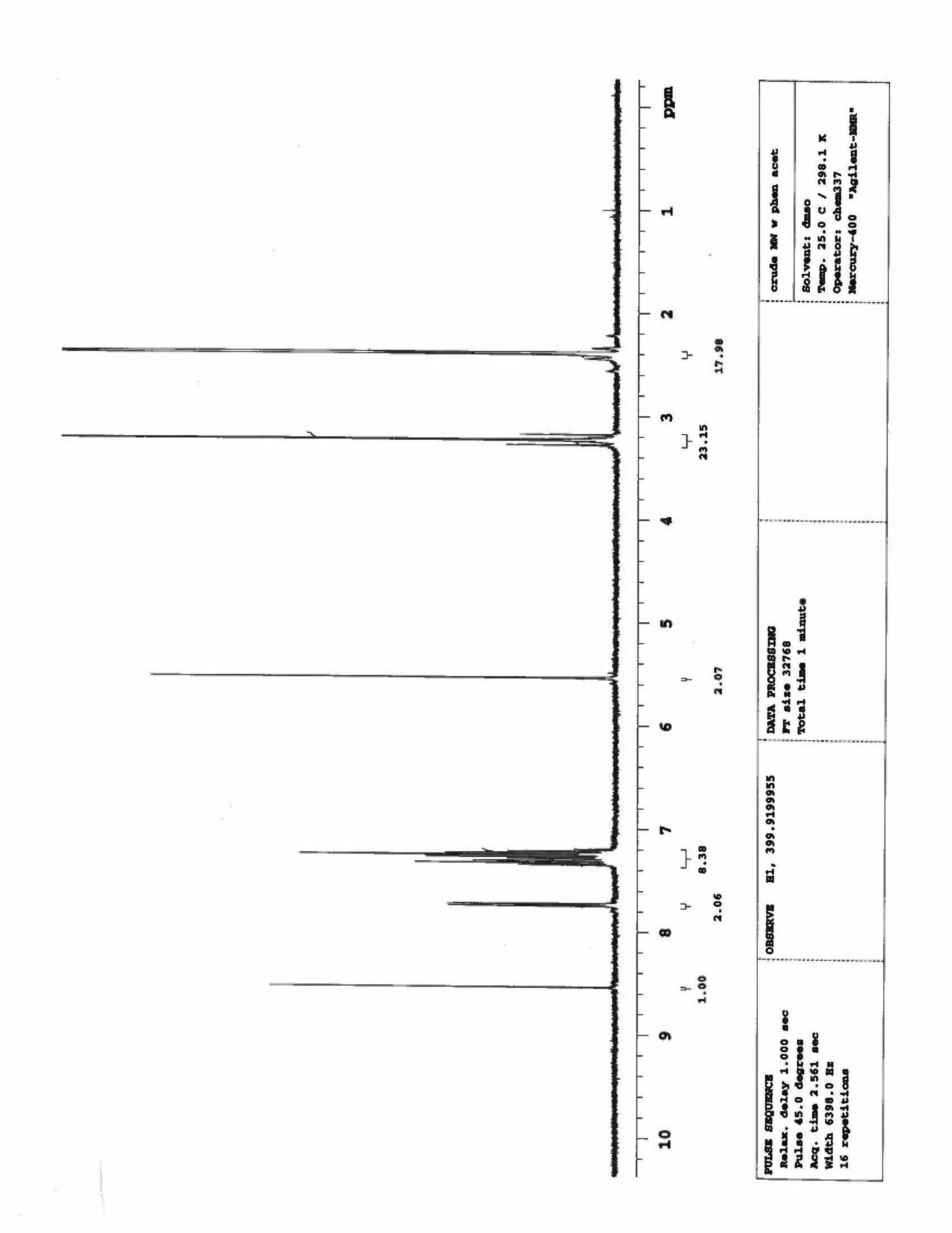

Microwave scale up of **1**.

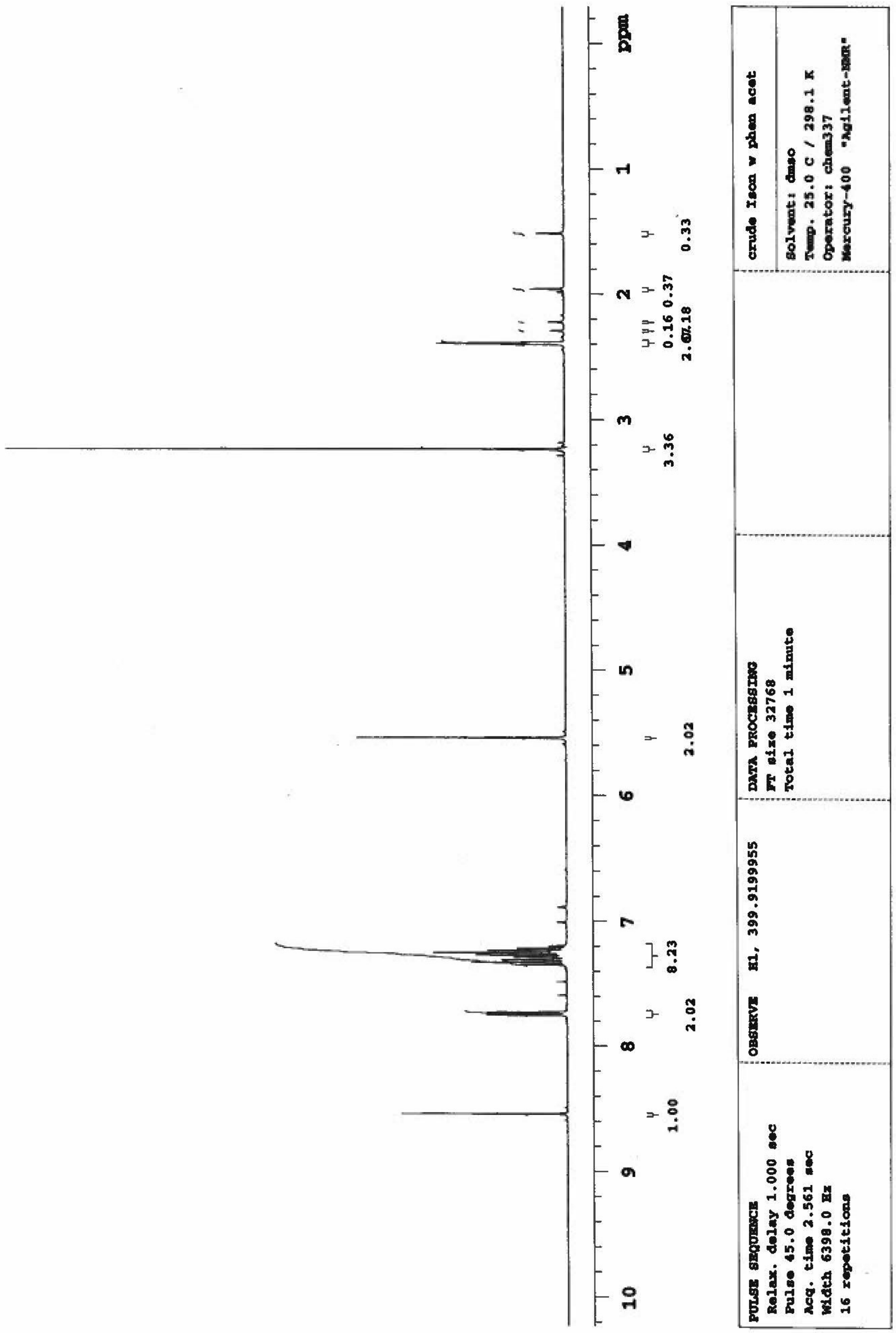

Solvent -free scale up of **1**.

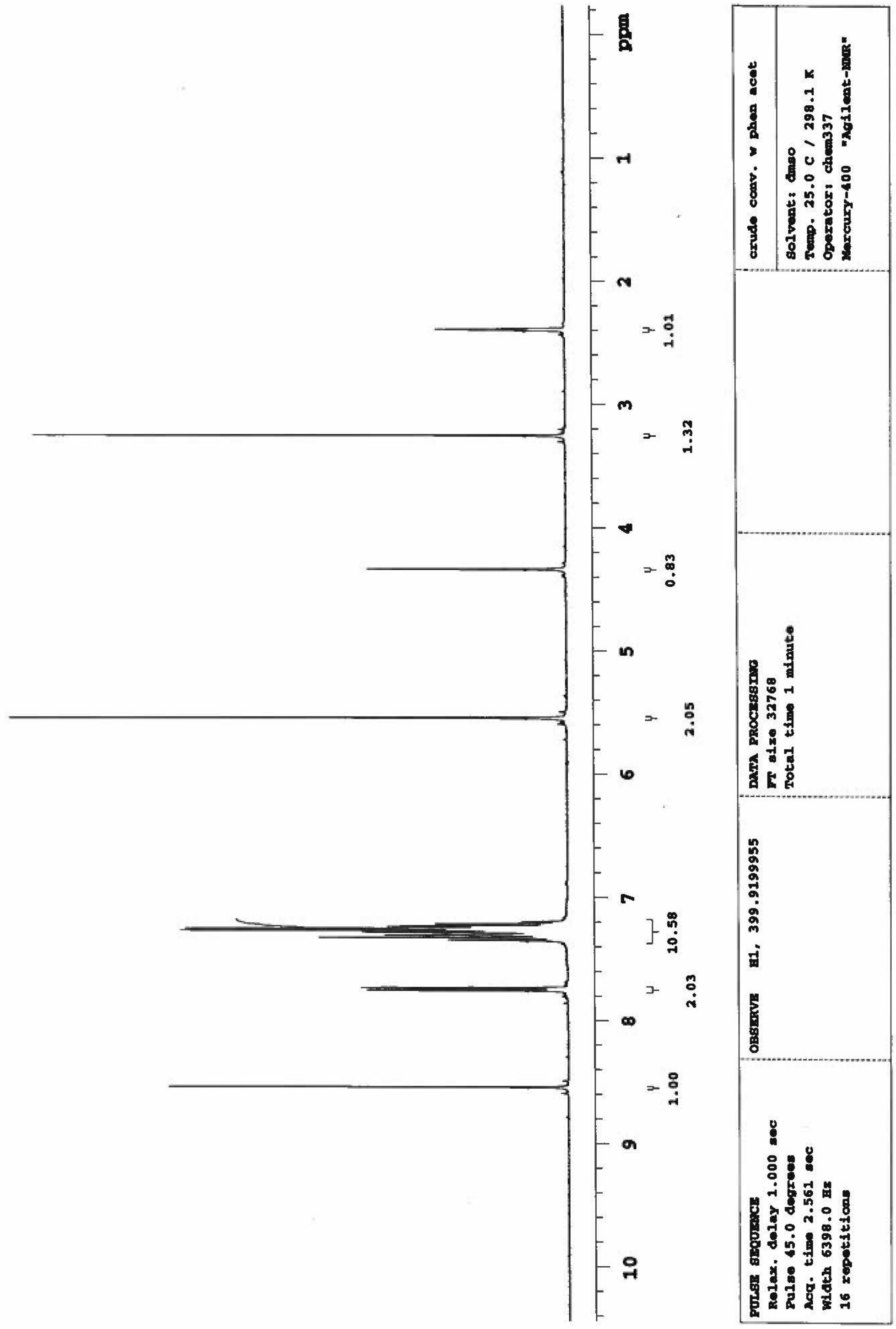

Conventional scale up of **1**.

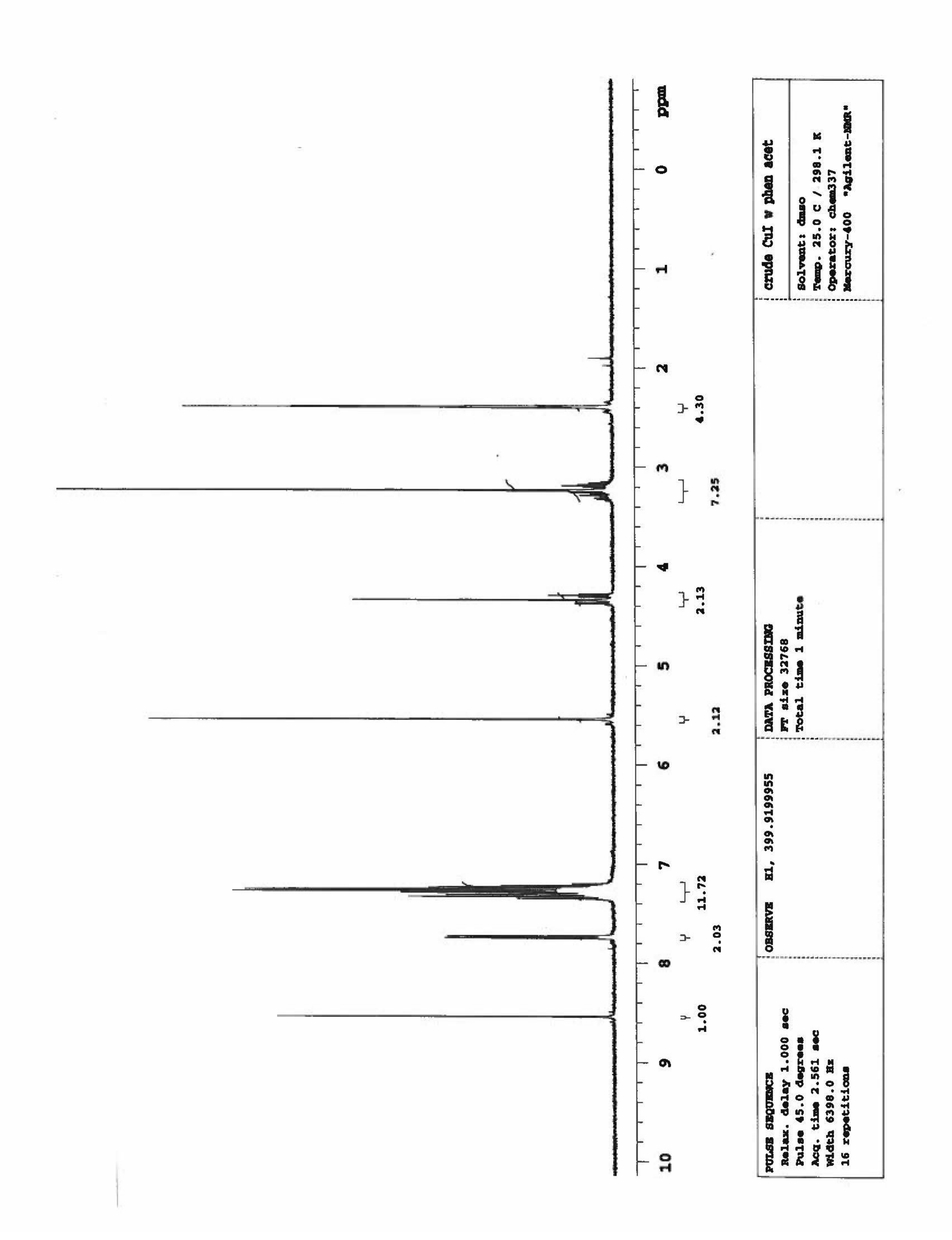

CuI in glycerol scale up of **1**.

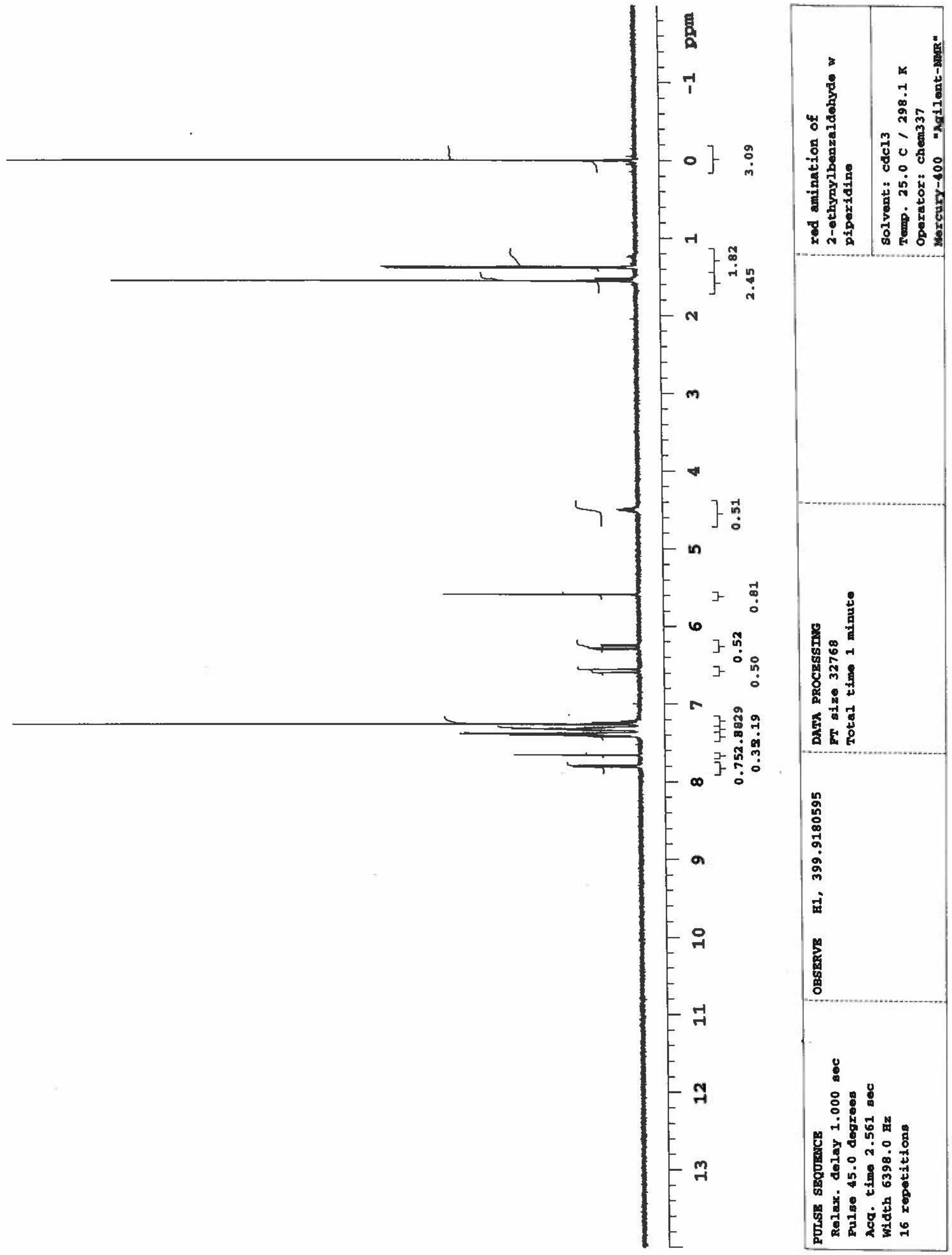

Solvent-free product **1** from equimolar reactants.

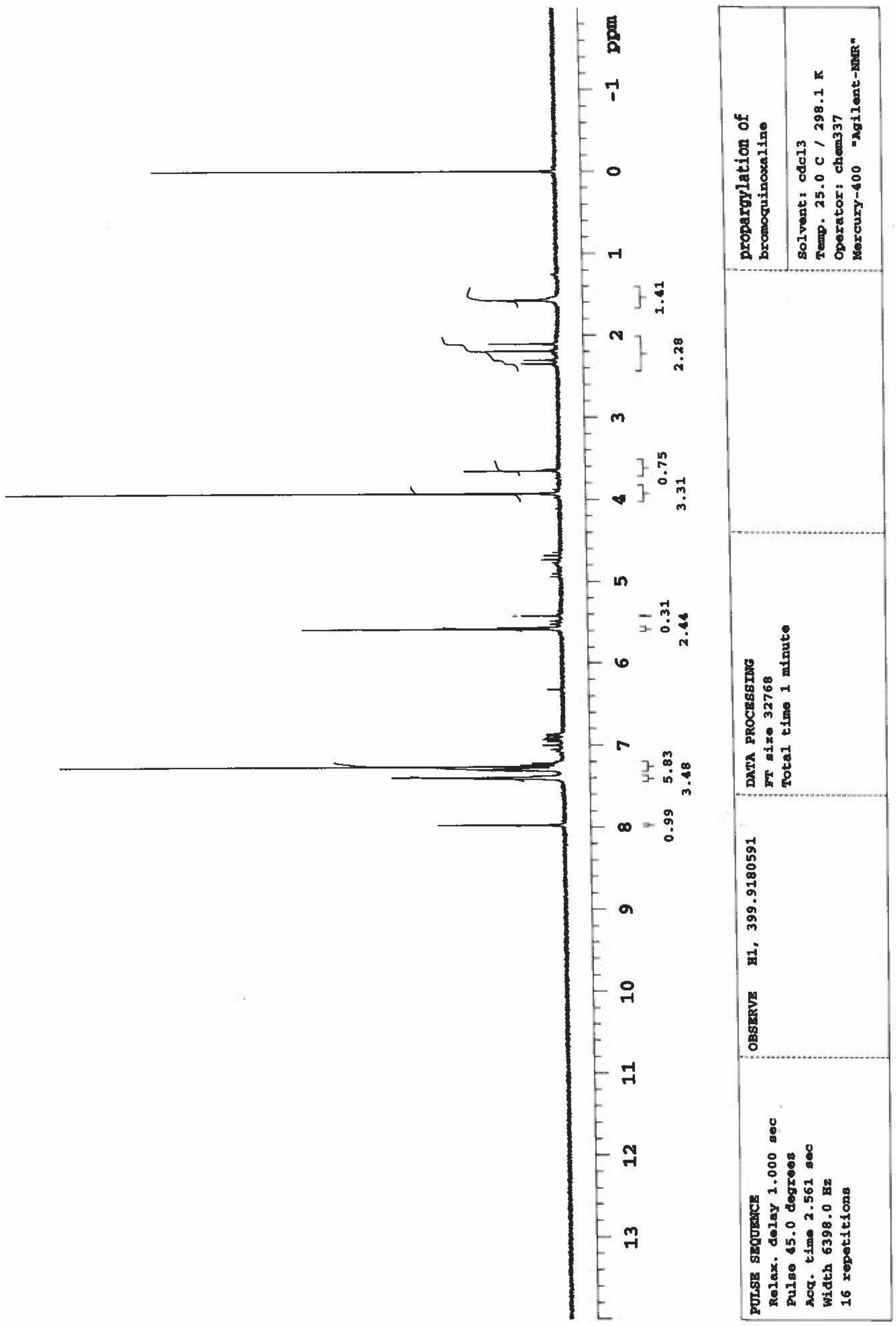

Solvent-free product **2** from equimolar reactants.

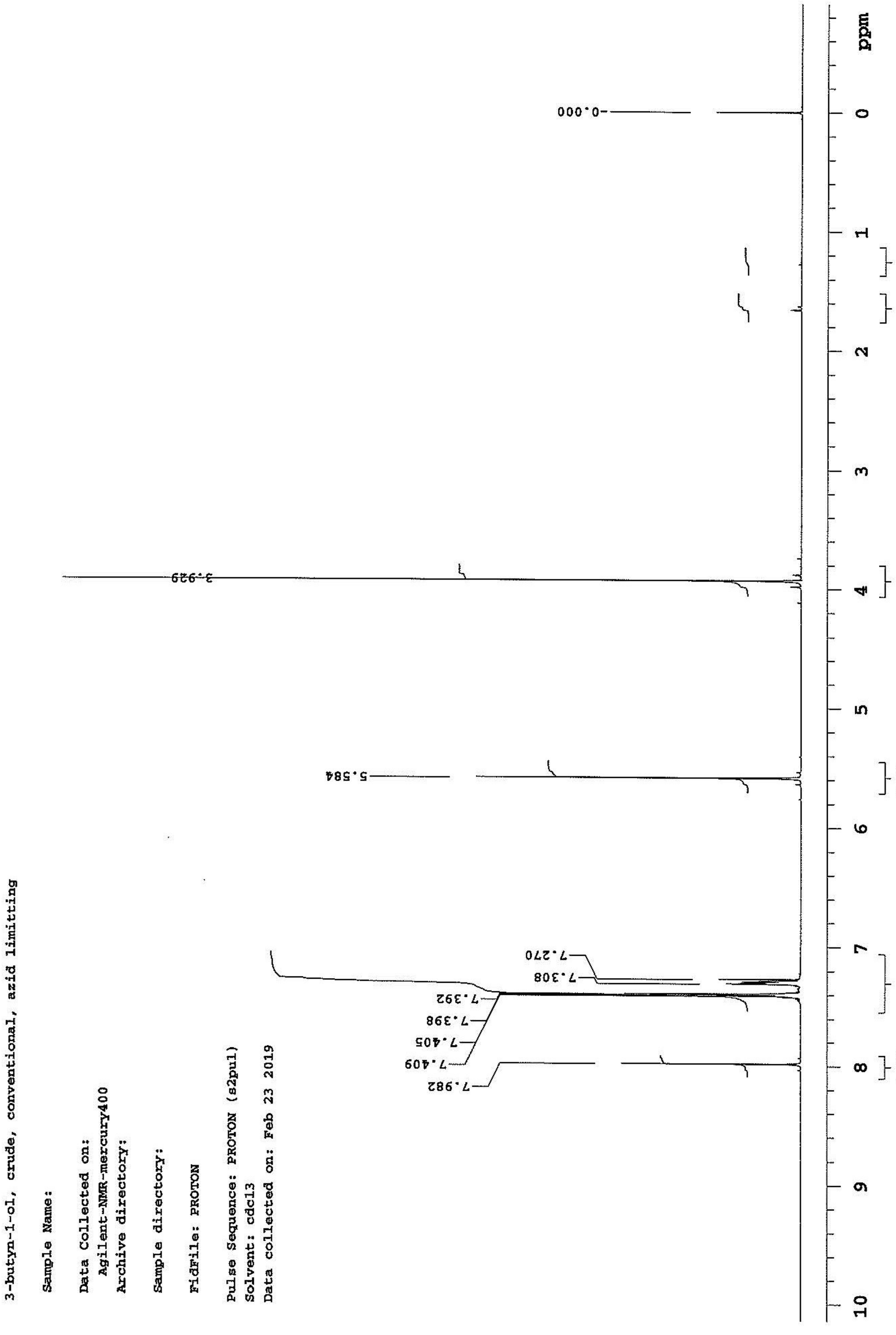

Conventional product **2** from 50% excess of methyl propiolate.

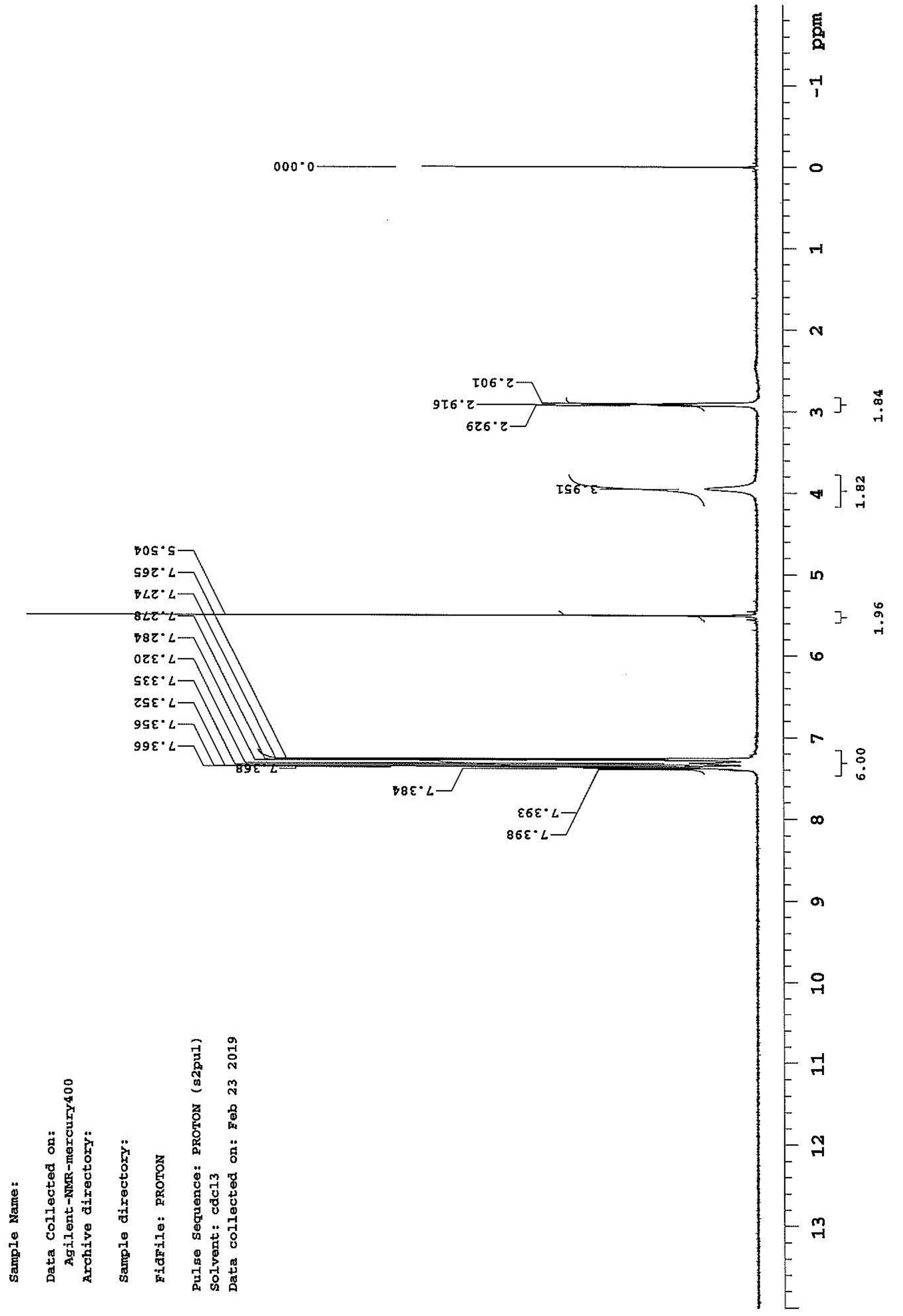

Conventional product **3** from 50% excess of 3-butyn-1-ol.

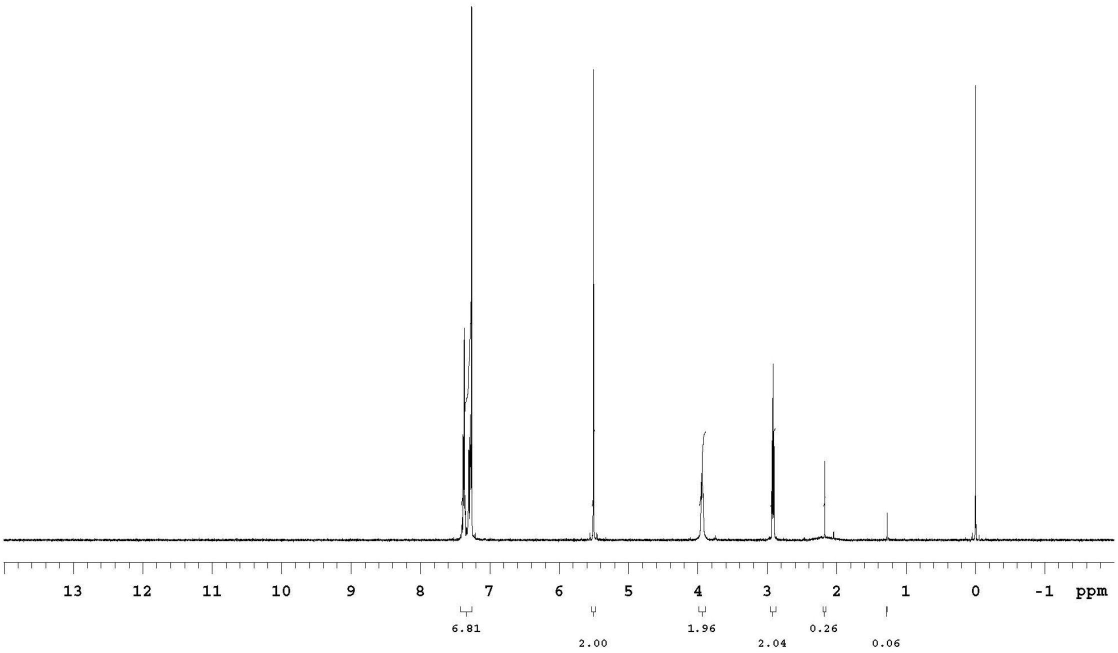

PULSE SEQUENCE<br>Relax. delay 1.000 sec<br>Pulse 45.0 degrees<br>Acq. time 2.561 sec<br>Width 6398.0 Hz<br>16 repetitions	OBSERVE H1, 399.9180595	DATA PROCESSING<br>FT size 32768<br>Total time 1 minute		Excess Alkyne, MW<br>3-Butyn-1-ol<br><br>Solvent: cdcl3<br>Temp. 25.0 C / 298.1 K<br>Operator: chem337<br>Mercury-400 "Agilent-NMR"

Microwave product **3** from 50% excess of 3-butyn-1-ol.

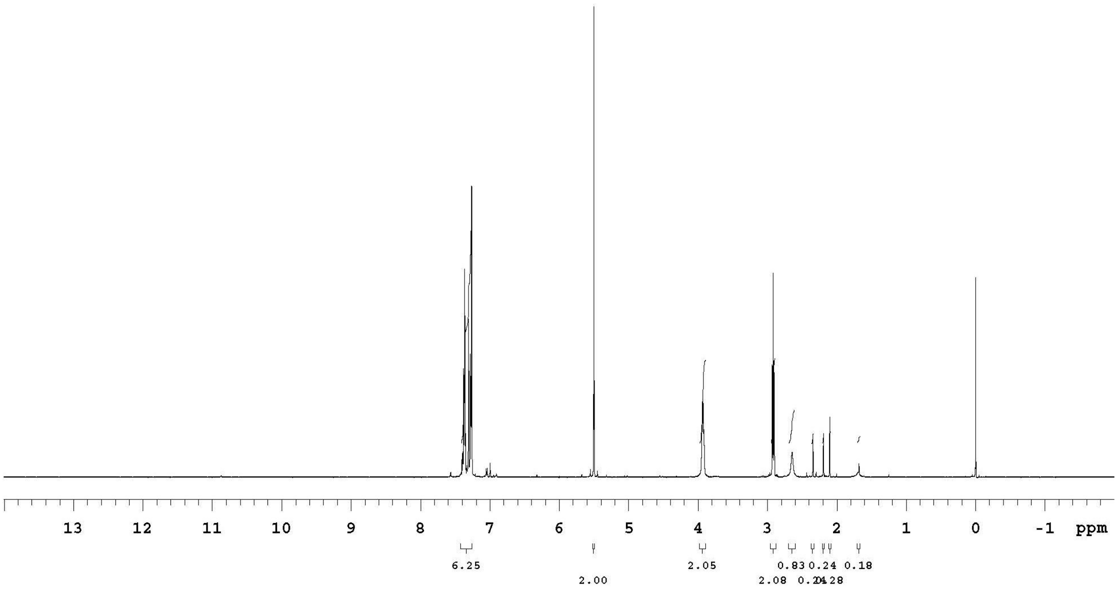

PULSE SEQUENCE<br>Relax. delay 1.000 sec<br>Pulse 45.0 degrees<br>Acq. time 2.561 sec<br>Width 6398.0 Hz<br>16 repetitions	OBSERVE H1, 399.9180588	DATA PROCESSING<br>FT size 32768<br>Total time 1 minute		Excess Alkyne, Ison<br>3-Butyn-1-ol<br><br>Solvent: cdcl3<br>Temp. 25.0 C / 298.1 K<br>Operator: chem337<br>Mercury-400 "Agilent-NMR"

Solvent-free product **3** from 50% excess of 3-butyn-1-ol.
